# Single Cell Proteolytic Assays to Investigate Cancer Clonal Heterogeneity and Cell Dynamics Using an Efficient Cell Loading Scheme

**DOI:** 10.1038/srep27154

**Published:** 2016-06-10

**Authors:** Yu-Chih Chen, Yu-Heng Cheng, Patrick Ingram, Euisik Yoon

**Affiliations:** 1 Department of Electrical Engineering and Computer Science, University of Michigan, 1301 Beal Avenue, Ann Arbor, MI 48109-2122, USA; 2 University of Michigan Comprehensive Cancer Center, 1500 E. Medical Center Drive, Ann Arbor, MI 48109, USA; 3 Department of Biomedical Engineering, University of Michigan, 2200 Bonisteel, Blvd. Ann Arbor, MI 48109-2099, USA

## Abstract

Proteolytic degradation of the extracellular matrix (ECM) is critical in cancer invasion, and recent work suggests that heterogeneous cancer populations cooperate in this process. Despite the importance of cell heterogeneity, conventional proteolytic assays measure average activity, requiring thousands of cells and providing limited information about heterogeneity and dynamics. Here, we developed a microfluidic platform that provides high-efficiency cell loading and simple valveless isolation, so the proteolytic activity of a small sample (10–100 cells) can be easily characterized. Combined with a single cell derived (clonal) sphere formation platform, we have successfully demonstrated the importance of microenvironmental cues for proteolytic activity and also investigated the difference between clones. Furthermore, the platform allows monitoring single cells at multiple time points, unveiling different cancer cell line dynamics in proteolytic activity. The presented tool facilitates single cell proteolytic analysis using small samples, and our findings illuminate the heterogeneous and dynamic nature of proteolytic activity.

90% of cancer-related deaths are caused by cancer metastases rather than the primary tumor^1^. Since proteolytic cleavage of extracellular matrix (ECM) proteins is essential in metastasis, the understanding of proteolytic activity can facilitate the design of new protease targeting drugs for clinical use^2^,^3^. To investigate proteases such as matrix metalloproteinases (MMP) and A Disintegrin And Metalloproteinases (ADAM), two important protease families in matric remodeling and growth factor shedding, researchers have developed protease sensitive fluorescent substrates based on fluorescence resonance energy transfer (FRET) mechanisms^4^,^5^. The fluorescence intensity of the dye increases when proteases cleave the amino acid-based substrate. As a result, the fluorescence intensity serves as a measure of proteolytic activity, enabling *in vitro* live-cell protease assays^5^.

Due to genetic and epigenetic instability in cancer (caused by environmental factors, faulty repair mechanisms, etc.), subgroups of cancer cells in a tumor can have very distinct phenotypes, and these differences in behavior pose great challenges to the treatment of cancer^6^,^7^. Recently, researcher demonstrated that the cancer invasion is driven by the cooperation of heterogeneous cancer cells. A “division of labor” between inherently invasive cells, which possess protease activity, and non-invasive cells can facilitate tumor invasion. This research shows the importance of cell heterogeneity in proteolytic activity for metastasis^8^,^9^. As dish based methods only provide information about the average behavior of bulk cells, single cell resolution methodologies are required to unveil the mystery of tumor heterogeneity. In addition, cell dynamics is another intriguing aspect in oncology^10^,^11^. The study of cell dynamics can dissect the cell heterogeneity in the time domain, which can be critical for both fundamental cancer modeling and protease-related clinical solutions^12^. For instance, different treatment strategies can be implemented if only a small subpopulation of cancer cells have constitutively high proteolytic activity rather than all the cells going through cycles of high and low activity stochastically^6^,^13^. In order to probe cell dynamics, the capability to track an individual single cell continuously is required^2^. As conventional dish based method do not provide methods for single cell tracking, single cell proteolytic activity dynamics has not previously been explored.

Thanks to their small sample handling capabilities, microfluidic technologies have already enabled single cell gene expression analysis, including real-time reverse transcription-polymerase chain reaction (RT-qPCR), digital PCR, and whole-transcriptome sequencing^14^,^15^,^16^. However, as proteases require enzyme activation to be functional, results may not reflect the true proteolytic cleavage activity^2^,^17^. Single cell western blotting allowing researchers to analyze proteins directly^18^, but it is a destructive process, allowing only a single time point to be measured. Some microfluidic technologies that incorporated the aforementioned protease sensitive fluorescent substrates were reported for probing proteolytic activity directly^19^, yet the existing tools have low cell loading efficiency while using small samples. However, since only a limited number of cells can be obtained from a variety of sources such as primary biopsies and microlavages, or when interfacing other microfluidic devices, high cell capture efficiency from low abundance samples is necessary. In continuous-flow microfluidics, most single cell isolation processes, such as hydrodynamic, micro-well-based cell settling or antibody-based capture, inevitably result in cell loss because of the dead volume and nature of the cell capture mechanisms, making these methods ill-suited to characterize small samples^20^,^21^,^22^,^23^. In addition, microwell-based systems have issues of media evaporation, reliable media exchange, and microwell isolation^21^. For droplet-based single-cell assays, washing, supplying media, and complete assay substrate exchange are challenging. Without an integrated method for droplet capture or tracking, droplet-based methods are not ideal for measuring the time dynamics of proteolytic activity^19^,^24^,^25^. Active capture mechanisms such as optical tweezers have low throughput and thus limit the utility of the technology^26^.

In this paper, we present a microfluidic proteolytic assay chip capable of capturing and isolating small cell samples and providing a robust methodology for media and reagent exchange. Using this platform, we investigated the heterogeneity that exists within cancer cell lines. Those that previously showed the importance of heterogeneity in invasion used a mix of cell lines to simulate innate heterogeneity; here we examined whether these characteristics are present within a single population and also their dynamic behavior. Additionally by integrating two separate microfluidic approaches, we successfully examined inter- and intraclonal proteolytic heterogeneity. To the best of our knowledge, this is the first attempt to explore the clonal heterogeneity and dynamics of single tumor cells.

## Results

### Single cell loading scheme

**Figure 1:**
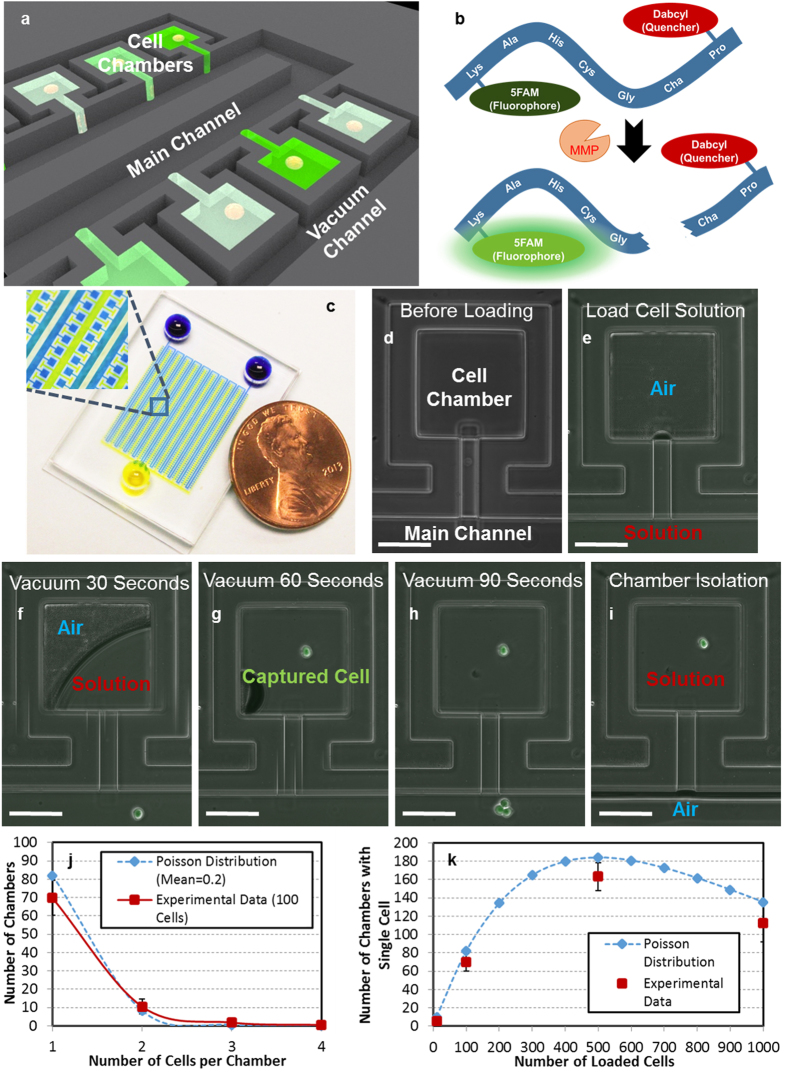
High-efficiency single cell capture scheme. (**a**) Schematic showing the single cell proteolytic assay. (**b**) The mechanism of the FRET-based sensing substrate. Before cleavage, the fluorophore is quenched. After cleavage, the substrate becomes brighter. (**c**) Photograph of a fabricated device. (**d–i**) The cell loading process: (**d**) empty chamber before loading, (**e**) loading the cell solution into the main channel, (**f**) solution partially fill the chamber after 30 seconds of applied vacuum (**g**) after 60 seconds of applied vacuum, one cell was captured, (**h**) after 90 seconds of applied vacuum, the chamber was completely filled with the solution, and (**i**) the cell solution in the main channel was driven downstream for further loading. (scale bar: 100 μm) (**j**) The number of chambers capturing 1, 2, 3, or 4 cells, when loading 100 cells into a 500-well device (N = 4 devices). (**k**) The number of chambers capturing exactly one cell with different number of loaded cells (N = 4 devices). The results match well with the Poisson distribution model.

The presented platform is composed of a main-channel, which transports the cell suspension and sensing fluorescent substrate, an array of 1,000 chambers for single cell capture, and a vacuum channel, which is used to drive the solution into the chambers (Fig. 1a). To measure the proteolytic activity, single cells and the commercially available FRET based substrate were loaded into the chambers. After loading, the chambers were isolated, and after the assay, the fluorescent intensity in the chamber indicates the activity of protease.

In order to facilitate high efficiency capture, a loading scheme has been developed that minimizes loading dead volume. In this scheme, the total volume of cell solution loaded is comparable to the total volume of the chambers (4 nL per chamber, ~4 μl total) and less than the total chip volume. For loading, a solution containing the cells of interest (a couple uL in volume) is pipetted into the inlet and fills the first few rows of the main channel (Fig. 1d,e). The solution does not fill the cell chambers due to surface tension effects and the air remaining in the chambers. Then, low pressure is applied to the vacuum channel that surround the cell chambers (Supplementary Fig. 1). As the material used for fabrication of this device (Supplementary Fig. 2), Polydimethylsiloxane (PDMS), has a high permeability^27^,^28^, the air can diffuses through the sidewalls. In this way, the vacuum pulls the air from the chamber, and the air in the chamber can be gradually replaced by the cell solution within 90 seconds (Fig. 1f–h, Supplementary Video 1). Once all the chambers within this first subsection are filled, the cell solution in the main channel can be pumped further downstream, and loaded into the next set of rows downstream (Fig. 1i, Supplementary Fig. 3). After several iterations of these steps, the entirety of the cell solution sample can be loaded into the chambers throughout the device. In this manner, the scheme can minimize the dead volume to achieve efficient use of small sample, even down to around 10–100 cells. After loading, the distribution of cells per chambers should follow a Poisson distribution. When the number of cells loaded is much smaller than the total number of the chambers, it is likely that the chambers will capture single cells. As a demonstration, we loaded 10 cells in a 500-chamber device, and 7 single cells were captured in the chambers (Supplementary Fig. 4). When we loaded 100 cells, the distribution matched well with the Poisson model (Fig. 1j). In cases where we have many cells, the number of cells loaded can be optimized using a Poisson model to achieve the highest capture rate (Fig. 1k). After loading is complete, we can isolate chambers for the proteolytic assay by pumping air into the main channel. The isolation can be released by flowing media into the main channel for cell culture, providing simple and robust valveless isolation (Supplementary Figs 5 and 6). Furthermore, the captured single cells were viable and proliferative even after 7 days of culture, indicating that the platform and the cell loading process do not affect cell viability and activity (Supplementary Fig. 7).

### Single cell proteolytic activity assay

To characterize the proteolytic activity of cells, a fluorescence resonance energy transfer (FRET) based substrate, composed of a FRET donor and quencher fluorophore, was used. The donor and the quencher are linked by amino acids, which can be cleaved by proteases (sequence: Dabcyl-Pro-Cha-Gly-Cys(Me)His-Ala-Lys(5FAM)-NH2). After cleavage, the distance between the pair increases, making quenching less efficient. Thus, the increase of fluorescent intensity can be used to indicate the proteolytic activity in the chamber^29^. First, we performed control experiments, verifying that fluorescent intensity increases with higher concentration of trypsin, which cleaves proteins, and longer assay time (Supplementary Figs 8 and 9). Also, the proteolytic activity of multiple breast cancer cell lines was measured in 96-well plates (Supplementary Fig. 10). These produced the expected results, validating the substrate and approach.

**Figure 2:**
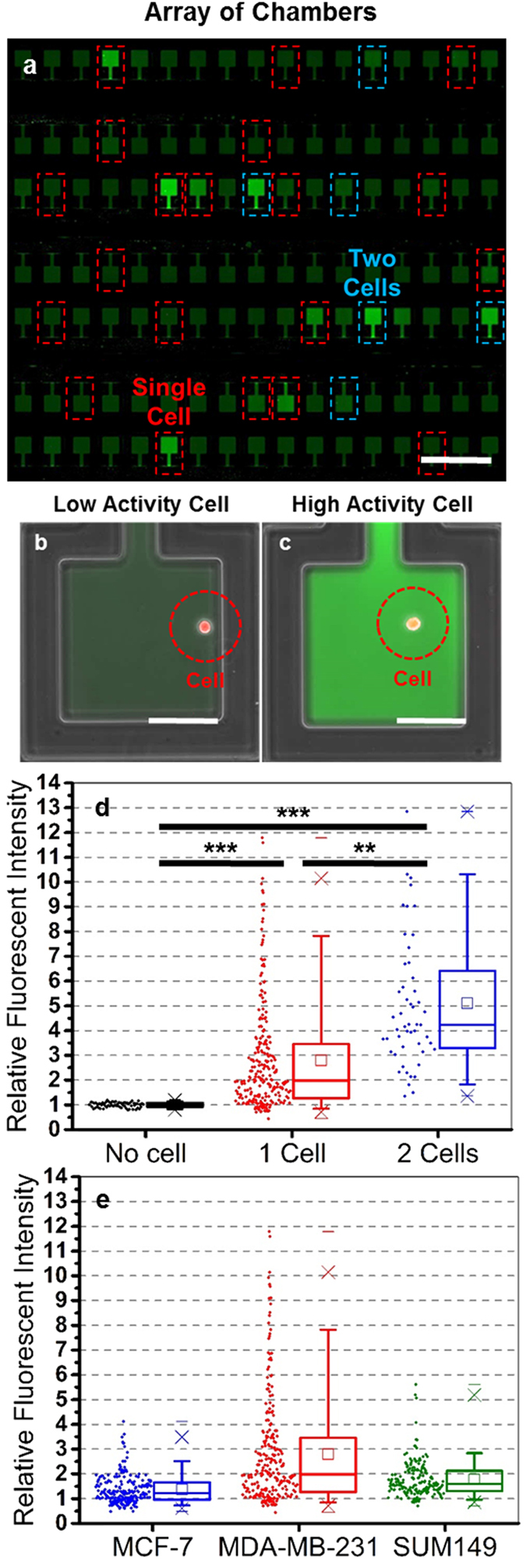
Single cell proteolytic assay. (**a**) An array of cell chambers. The chambers circled with red dashed lines captured one cell, and the ones circled with blue dashed lines captured 2 cells. (scale bar: 1 mm) (**b,c**) Representative case of (**b**) a low proteolytic activity cell and (**c**) a high proteolytic activity cell. (scale bar: 100 μm) The fluorescent intensity reflects the proteolytic activity. (**d**) A box plot of the proteolytic activity of MDA-MB-231 cells; each dot represents one data point. The chambers containing 1 cell have significant higher fluorescent intensity than the empty chambers, and the chambers containing two cells are even brighter. (N ∼ 200 cells for 0 and 1 cell per chamber, N ∼ 50 for 2 cells per chamber) (**e**) A box plot of the proteolytic activity of MCF-7, MDA-MB-231, and SUM149 cells. A portion of MDA-MB-231 cells have very high activity, while the cells are more homogeneous for two other cell lines. (N ∼ 200 cells for all three cell lines). *Refers to P < 0.05, **Refers to P < 0.01, and ***Refers to P < 0.001.

For quantification of the proteolytic activity of single cells, we loaded single cells into the chambers as describe above. After that, the FRET substrate was loaded into the chamber and the chamber was isolated (Supplementary Fig. 11). Figure 2a–c shows an array of chambers containing single cells and the enlarged view of cells with high and low proteolytic activity. To cancel the background noise, we normalize the fluorescent intensity using the empty chambers on the same chip to minimize the device to device variation. After normalization, the fluorescent intensity of chambers containing 1–2 cells are significantly higher than that of empty chambers (Fig. 2d). When we characterizing three different cell lines: MCF-7, MDA-MB-231, and SUM149, different patterns of heterogeneity were observed (Fig. 2e). The distribution of MDA-MB-231 has a long tail of highly active cells, while the SUM149 and MCF-7 cells are more uniform (Supplementary Fig. 12). Since bulk assays only report the average behavior within a population, obscuring the contribution of highly active sub-populations, no previous study of cancer cell protease activity has successfully observed these heterogeneous behavioral patterns.

### Intraclonal heterogeneity in proteolytic activity

When quantifying the proteolytic activity of single cells in the previous experiment, we observed the innate heterogeneity expressed within a population. However, several recent studies have expounded the importance of microenvironmental regulation to cell heterogeneity^30^,^31^,^32^. A challenge then arises when trying to differentiate the contributions of the microenvironment from the innate heterogeneity of the cell population. To alleviate this complication, we can analyze microenvironmental effects on clonal populations. A microfluidic platform previously developed in our group, provides an ideal solution by facilitating the creation of single cell-derived (clonal) spheres (Supplementary Fig. 13)^33^. The location of the cells within the sphere provides different microenvironmental cues that modulate protease activity of the clonally identical cells.

**Figure 3:**
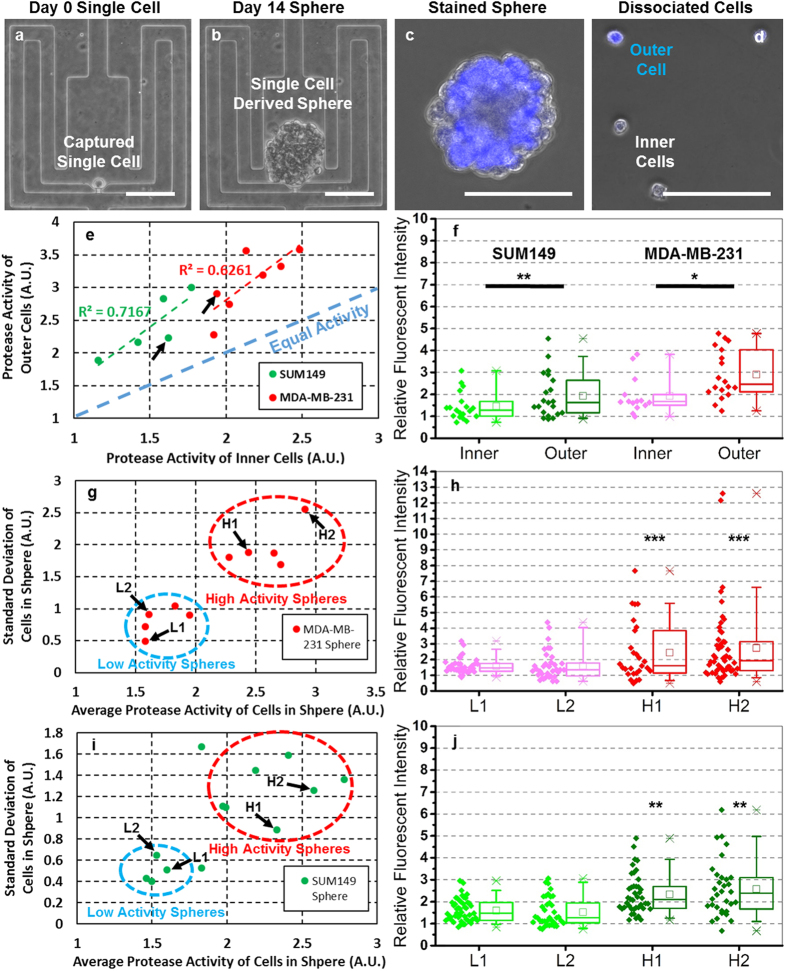
Intraclonal and interclonal heterogeneity of proteolytic activity using single cell derived cancer spheres. (**a–d**) The process of MDA-MB-231 clonal sphere formation and analysis (scale bar: 100 μm) (**a**) cells captured on day 1 on non-adherent substrate, (**b**) single cells form sphere after 14 days, (**c**) sphere staining (blue cell tracker) to distinguish the outer and inner cells and (**d**) the dissociation of spheres for single cell analysis. (**e**) The proteolytic activity of inner and outer cells, the x-axis is the average activity of inner cells and y-axis is the average activity of outer cells. Red and green dash line show the correlation between inner and outer cells by regression. The blue dash line is the equal activity line, showing that the average outer cells have higher activity than inner ones for all spheres. (N = 5 spheres or around 150 cells for SUM149, N = 7 spheres or around 200 cells for MDA-MB-231) (**f**) The box plot of proteolytic activity of inner and outer cells from representative SUM149 and MDA-MB-231 spheres (indicated in the previous figure by black arrows). (N ∼ 20 cells for all conditions). (**g**) The average (x-axis) and standard deviation (y-axis) of proteolytic activity of MDA-MB-231 spheres. The spheres are clustered into two categories (high and low) based on protease activity. (N = 10 spheres). (**h**) The box plot of two representative MDA-MB-231 spheres from high and low activity categories (indicated in the previous figure by black arrows). (N ∼ 40 cells for all spheres). (**i**) The average (x-axis) and standard deviation (y-axis) of proteolytic activity of SUM149 spheres. The spheres are clustered into two categories (high and low) based on protease activity. (N = 14 spheres). (**j**) The box plot of two representative SUM149 spheres from high and low activity categories (indicated in the previous figure by black arrows). (N ∼ 40 cells for all spheres). *Refers to P < 0.05, **Refers to P < 0.01, and ***Refers to P < 0.001.

For single cell derived sphere formation, a cell suspension was loaded into the device and single cells were captured at the capture site (Fig. 3a, Supplementary Videos 2 and 3)). As the bottom surface of the platform was coated with polyHEMA, a non-adherent polymer (Supplementary Fig. 14), the single cell grows in suspension to form a clonal sphere (Fig. 3b, Supplementary Fig. 15). After 14 days, we dissembled the device to retrieve the spheres. Current research suggests that we should expect different protease activity at the edge of a tumor as compared to the center^32^, as cells at the edge of the tumor must remodel the surrounding ECM in order facilitate further tumor growth and spread. In order to examine this phenomena in a small scale model, we demonstrated the differences in protease activity between inner and outer cells in a harvested sphere. Cell tracker was introduced into the device to stain the spheres but was washed away after only 3 minutes of incubation. This short incubation time combined with the limited diffusion within the sphere resulted non-uniform staining of the cells in the sphere (i.e. cell fluorescence on the sphere periphery was considerably brighter than that in the center) (Fig. 3c). Then, the sphere was dissociated into single cells by trypsin, and the difference in fluorescent intensity was preserved (Fig. 3d)^34^. Using the presented method, we demonstrated the intraclonal heterogeneity (caused by microenvironmental effects) of two cell lines: MDA-MB-231 and SUM149 (Fig. 3e). For all the spheres we tested, the average proteolytic activity of outer cells is higher than that of inner cells. In a representative sphere (Fig. 3f), outer cells have wide dynamic range of activity, while the all inner cells only show low protease activity. Using a mixture of inner and outer cells from different single cell derived spheres leads to less significant results as the innate interclonal differences in proteolytic activity are comparable in magnitude (Supplementary Fig. 16). The results support that the internal cells in a tumor are inactive in proteolytic activity^2^.

### Interclonal heterogeneity in proteolytic activity

Although we observed intraclonal heterogeneity caused by microenvironmental differences, the activities of inner and outer cells from the same sphere are highly correlated as well (Fig. 3e). To further examine this phenomena, we characterized more single cell derived spheres of MDA-MB-231 and SUM149 cells (Supplementary Figs 17 and 18). When we mapped the MDA-MB-231 spheres on a 2D plot using the average activity as the x-axis and standard deviation within sphere as the y-axis, two subtypes of spheres clustered: those with low activity and variation and those with both higher average activity and variation (Fig. 3g). The single cell activities of four representative MDA-MB-231 spheres are shown in Fig. 3h. SUM149 spheres follow similar trend (Fig. 3i,j). As we analyzed cells from different spheres using different devices, it was also important to examine whether the variation was caused by the device-to-device variation. We found that the devices loaded with bulk cells have significantly lower variation than those loaded clonal cells (Supplementary Fig. 12), indicating the heterogeneity observed was likely a result of the sphere culture and not the devices themselves. In addition, we plotted the sphere size (related to proliferation rate) versus the proteolytic activity of its dissociated cells (Supplementary Fig. 19). Surprisingly, we found that though larger spheres have more cells, the size of sphere has no correlation with single cell proteolytic activity. These results demonstrate the capability of presented approach to dissect the intraclonal and interclonal heterogeneity of cell lines.

### Dynamics of proteolytic activity

**Figure 4:**
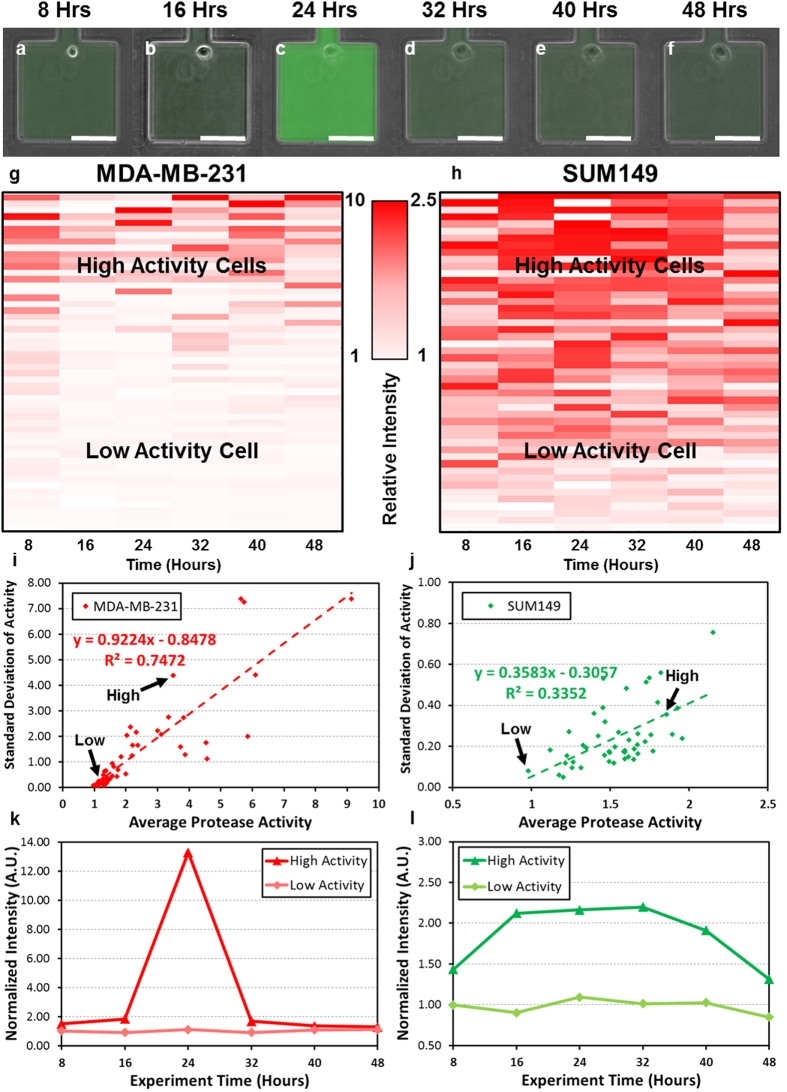
Dynamics of single cell proteolytic activity. (**a–f**) The dynamic activity of a MDA-MB-231 cell: (**a**) 8 hours after loading, (**b**) 16 hours, (**c**) 24 hours, (**d**) 32 hours, (**e**) 40 hours and (**f**) 48 hours. (scale bar: 100 μm) (**g**) The dynamics of the MDA-MB-231 cells plotted in a heat map. Red color represents high activity (relative fluorescent intensity: 10), and white color represents low activity (relative fluorescent intensity: 1). The cells were sorted based on the summation of the average and the standard deviation of their proteolytic activity. (N ∼ 50 cells for all 6 time points). (**h**) The dynamics of the SUM149 cells plotted in a heat map. Red color represents high activity (relative fluorescent intensity: 2.5), and white color represents low activity (relative fluorescent intensity: (**1**)). The cells were sorted based on the summation of the average and the standard deviation of their proteolytic activity. (N ∼ 50 cells for all 6 time points). (**i**) The average (x-axis) and standard deviation (y-axis) of proteolytic activity of MDA-MB-231 cells at different time points. Red dashed line shows the regression of the correlation between average and standard deviation. (**j**) The average (x-axis) and standard deviation (y-axis) of proteolytic activity of SUM149 cells at different time points. Green dash line shows the regression of the correlation between average and standard deviation. (**k**) Example dynamics of representative high activity and low activity MDA-MB-231 cells (indicated in (**i**) by black arrows). (**l**) Example dynamics of representative high activity and low activity SUM149 cells (indicated in (**j**) by black arrows).

After characterizing the heterogeneity between cells, we next investigated the variation of proteolytic activity of the same cell at different time points (dynamics). As the time constant of protein translation is several hours^10^, we measured single cell protease activity every 8 hours for 2 days. These time points provide sufficient time for dye diffusion and cell recovery between assays as well (Supplementary Fig. 20). Figure 4a–f show the activity of the individual cells at different time points, and the cells were clustered based on dynamics in the heat maps (Fig. 4g,h). Different dynamics between MDA-MB-231 and SUM149 were observed. The MDA-MB-231 had wide dynamic ranges of activity and sharp short pulse of high activity, causing high variation between time points (Fig. 4i). We were concerned that the sharp changes were the result of cell divisions, but when cells were monitored for division events, the data show no difference of activity before and after cell division takes place (Supplementary Fig. 21). Compared to the activity of MDA-MB-231, the activity of SUM149 cells changes gradually and slowly, resulting in low variation between time points (Fig. 4j). The representative cases of two cell line are plotted in Fig. 4k,l. In addition to demonstrate the cell stability on-chip, we verified that the average proteolytic activity of the single cell population remained stable at all time points (Supplementary Fig. 22).

## Discussion

The regulation of proteases is a key issue in cancer metastasis. Although proteolytic assays can be performed in a conventional (dish based) manner, the presented single cell approach provides three advantages over conventional approaches: (1) the capability to handle small samples (~10–100 cells), (2) the potential to investigate intraclonal and interclonal heterogeneity, and (3) ability to monitor and track individual cells over time. Conventional approaches need hundreds of thousands of cells for an assay. A patient biopsy sample may be barely enough for a single trial. Though hydrodynamic cell capture schemes can elegantly position single cells and achieve high (~80–90%) single cell capture rates (number of captured cells/number of chambers), the cell capture efficiency (number of captured cells/number of cells used) is typically lower (<10%). Two fundamental limitations are: (1) cells are lost in the dead volume that never flows into the device and (2) continuous flow in the hydrodynamic scheme inevitably carries a portion of the cells to the outlet without the cells being captured. The presented platform drives all the sample into the chambers to achieve a negligible dead volume, and all loaded cells will be retained in the chambers. As a result, we can achieve a high (>50%) capture efficiency, even for small samples. Compared to the approaches using open micro-well array^35^, the presented approach has advantages that include (1) easy and robust media/reagent exchange, (2) enclosed microfluidics to minimize the evaporation, and (3) reliable isolation between chambers. As such, this single cell enzymatic assay platform specializes in the single cell analysis of small samples, something not possible in previous approaches.

Using the platform, we can easily investigate the cellular heterogeneity that often complicates cancer treatment and analysis. Previous studies have used mixtures of different cell lines to mimic the *in-vivo* heterogeneity and showed that heterogeneity in and of itself contributes to invasion^8^,^9^. However, in these experiments, we demonstrated these differences exist within single lines. MDA-MB-231 cells show a wide distribution of proteolytic activity, while SUM149 are more homogeneous and less active. The high proteolytic activity and their innate heterogeneity may contribute to these cells stronger metastatic potential as compared to SUM149.

As suggested in the literature, microenvironment had a large effect on proteolytic activity. Sphere formation provides one approach to look at these effects as the location of the cells within a sphere provides different cues that modulate protease activity. By beginning with single cell derived spheres, the differences in activity can be attributed to these cues alone. As we have a limited number of clonal cells from a sphere, it is necessary to be able to analyze small samples. First, we found that within a 3D sphere, the inner cells have lower proteolytic activity, while the outer cells are highly active. This result verifies the heterogeneous nature of proteolytic activity in a tumor sphere and supports the importance of microenvironmental effect during tumor growth^32^. Located at different positions within a sphere, the descendant cells from the same progenitor can have significantly different proteolytic activity. Although the cells in the same sphere are heterogeneous, the proteolytic activities of inner cells and outer cells are still correlated. This heterogeneity was observed despite the fact that cancer cell lines are believed to be more homogeneous than primary patent samples. For both MDA-MB-231 and SUM149 cells, the clones can be classified as low activity or high activity spheres. The cells in high activity clones are highly heterogeneous, while those in low activity clones have homogeneously low activity. We also tried to correlate the sphere size, which is proportional to the cell proliferation rate, and the proteolytic activity. Surprisingly, we didn’t observe correlation between the size and the average proteolytic activity, indicating that the regulation of proteolytic activity seems to be independent of the cell proliferation and cell cycle. As most cancer cell heterogeneity studies are based on bio-markers using fluorescence-activated cell sorting (FACS), the next step to integrate our findings into the literature more thoroughly would be to investigate the relationship between markers and the proteolytic activity. One possible study would be to investigate the relationship between protease activity and cancer cell “stem-ness”. In breast cancer, researchers have identified a rare subpopulation of tumorigenic cancer stem-like cells (CSC), often thought to be the source of drug resistance and tumor metastasis^36^,^37^,^38^. These populations are typically identified by ALDH or CD44+/CD24−37,38. As proteases are required for cancer cell invasion, the correlation may clarify the roles of different sub-populations in tumor metastasis.

Though we successfully investigated inter and intra- clonal heterogeneity, it was still not known whether high proteolytic activity is the intrinsic characteristic of a particular cell or if the proteolytic activity of single cell follows a stochastic pattern, changing with time. Thus, we monitored the proteolytic activity of the same cell every 8 hours to understand its dynamics. The preliminary data suggest that the proteolytic activity of MDA-MB-231 seems to follow a stochastic pattern. The same cell can have wide (10 times) dynamic range of proteolytic activity at different time points, and the activity can increase and decrease rapidly within 8 hours. We first suspected the correlation between cell cycle and the proteolytic activity; however, when we examined the chambers with proliferating cells, we found that the proteolytic activity remains stable before and after cell division. Also, no difference can be found between proliferating and non-proliferating cells. This supports that the previous finding that proliferation rate seems to be independent of its proteolytic activity. Compared to MDA-MB-231, the proteolytic activity of SUM149 changes slowly. The elevated activity are maintained for 24 hours or longer, so the standard deviation of activity at different time points is significantly lower than that of MDA-MB-231 cells. This difference indicates that the protease regulating mechanisms of two cell lines are possibly quite different. These types of valuable information can be easily hidden when averaging activities of many cells are examined using dish based approaches. This data shows that the single cell analysis approached present here provides a novel tool to unveil the enzymatic activity pattern in time domain, enabling more mechanistic studies as the next step.

## Methods

### Microfluidic chip design and fabrication

Each microfluidic chip contains 1000 chambers (other numbers, such as 350 or 500, of chambers can be used depending on the size of samples) with 4 nL (200 μm × 200 μm × 100 μm) volume capacities. The 4 nL chamber volume was selected in order to balance engineering trade-offs. Though a larger chamber size would be favorable for cell survival due to a larger nutrient supply, the proteolytic enzyme activity in a single cell would result in a lower concentration. This would reduce the limit-of-detection and is, thus, undesirable. The main channel was designed to be 200 μm in width. This allows the cells to be transported into the cell chambers and also ensures sufficient proteolysis sensing substrate near the entrance of each chambers for diffusion into the chambers.

The microfluidic chip is composed of patterned PDMS (Polydimethylsiloxane, Sylgard 184, Dow Corning) bonded with a glass substrate. The PDMS layer is fabricated though a soft lithography process. The master mold is built by patterning 100 μm thick SU8 (Microchem) for defining the channel geometry on a silicon wafer using photolithography. After curing the PDMS on the mold, the PDMS and glass slides are bonded after oxygen plasma surface treatment. Detailed protocols for mold and device fabrication are available in Supplementary Notes 1 and 2.

### Cell culture

Several breast cancer cell lines such as MDA-MB-231, SUM149, MCF7, and T47D were cultured for the proteolytic assay cell experiments. MDA-MB-231 cells were obtained from Dr. Gary Luker’s Lab (University of Michigan, MI, USA). SUM149, MCF-7, and T47D cells were obtained from Dr. Max Wicha’s Lab (University of Michigan, MI, USA). MDA-MB-231, MCF- 7cells were cultured in DMEM (Gibco 11965) with 10% FBS (Gibco 10082) and 1% penicillin/streptomycin (Gibco 15070). SUM149 cells were cultured in the F-12 based media (Ham’s F12 (Gibco 11765) supplemented with 1% penicillin/streptomycin (Gibco 15070), 5 ug/mL insulin (Sigma I6634), 1 ug/mL hydrocortisone (Sigma H4001), 5% FBS (Gibco 10082). T47D cells were cultured in RPMI (Gibco 11875) with 10% FBS (Gibco 10082) and 1% penicillin/streptomycin (Gibco 15140). All the cells were cultured in polystyrene culture dishes and passaged when cells reached over 80% confluency in the dish.

### Bulk proteolytic activity assay

Before performing single cell proteolytic activity assays, we performed the bulk proteolytic activity assays in 96-well plates as a control. Trypsin without phenol (Gibco 15400), PBS, and the fluorescent substrate was mixed together to achieve trypsin concentration ranges from 500 μg/mL to 0.5 μg/mL, with a constant 20 μM of fluorescent proteolysis sensing substrate (BioZyme, PEPDAB008m001 Peptide, Fluorescent, Dabcyl, for MMPs, Dabcyl-Pro-Cha-Gly-Cys(Me)His-Ala-Lys(5FAM)-NH2, Purity >92%, MW = 1388.3 g/mol). The protease sensing substrate used in the paper (PEPDAB008) has stronger response to MMP13 and MMP9, and its complete specificity constant table can be downloaded from the official BioZyme website. 100 μL of each solution was pipetted to each well in the 96-well plate for measurement. After 30 minutes, the fluorescent intensity was measured using a fluorescent microscope, and the intensities were normalized to the intensity from the fluorescent substrate mixed with PBS (negative control). Multiple time (30 minutes, 60 minutes, 120 minutes, and 180 minutes) points were measured to plot the dynamics of the reaction as well. For the bulk cell proteolytic activity cell assay, one thousand and 10 thousand cells were seeded (in serum containing culture media) in the 96-well plate on day 0. One day after cell plating, the cells had adhered on the substrate. Then, the cells were washed with PBS 3 times to remove all residual serum, which can interfere with the result. Then, 100 μL of the 20 μM fluorescent substrate was added to the wells. After 1 hour of incubation time, the 100 μL solution was transported to another 96-well plate for fluorescent measurement. This would prevent the light absorption by the cells on the bottom of the wells to affect the readout. The fluorescent intensity was measured using a fluorescent microscope, and the results were normalized to intensity of the negative control.

### Microfluidic cell loading

The microfluidic chips were sanitized by UV radiation prior to cell loading to ensure aseptic conditions. For small sample loading, a solution containing the cells of interest (a couple uL in volume) was pipetted into the inlet and filled the first few rows of the main channel using the negative pressure generated by a Pasteur pipette bulb (~1000 Pa). Then, negative pressure (~0.1 atm) was applied to the vacuum channel that surround the cell chambers (Supplementary Fig. 1). As the material used for fabrication of this device (Supplementary Fig. 2), Polydimethylsiloxane (PDMS), has a high permeability, the air can diffuse through the sidewalls. In this way, the vacuum pulled the air from the chamber, and the air in the chamber was gradually replaced with the cell solution within 90 seconds (Supplementary Video 1). Once all the chambers within this first subsection were filled, the cell solution in the main channel was pumped further downstream, and loaded into the next set of rows downstream (Supplementary Fig. 3). After several iterations of these steps, the entirety of the cell solution sample were loaded into the chambers throughout the device. In this manner, the scheme could minimize the dead volume to achieve efficient use of small sample, even down to around 10–100 cells.

If we have large amount of cells in the sample, it is important to control the cell concentration to optimize the single cell capture rates. Modeling single cells in 4 nL chambers yields an ideal cell concentration of around 250 k cells/mL, which is the cell concentration chosen for cell loading. In this case, 50 μL of the cell solution was pipetted into the device inlet, negative pressure (~1000 Pa) generated by a Pasteur pipette bulb was first applied to the outlet to drive the solution into the main channel. Negative pressure generated by a vacuum pump (~0.1 atm) was then applied to the vacuum channel for two minutes to drive cell solution into the culture chambers and achieve single cell capture. The distribution of chambers with 0, 1, 2, and 3 cells follows a Poisson distribution. After the cell loading, the cell solution was removed from the device inlet and outlet. To wash away the residual cells in the main channel that could affect the proteolytic assay, fresh culture media was added to the inlet and negative pressure generated by the vacuum pump was applied at the outlet for ten seconds to wash all the cells in the main channel to the outlet. The washing process was repeated three times and all media was removed from the outlet. Finally, fresh media was added to the device inlet for cell incubation.

### Microfluidic single cell proteolytic assay

After the cell loading, the microfluidic devices were placed in an incubator (Nuaire) for 6 hours before the experiments began. The temperature was maintained at 37 °C with 5% CO_2_ in humidified air. The substrate for proteolytic assay was diluted to 20 μM in PBS. To load the substrate into the cell chambers, the media in the inlet and outlet was removed and replaced with the substrate solution. Negative pressure generated by a pipette bulb was again applied to the outlet to drive the substrate solution through the main channel, to allow the substrate to diffuse into the cell chambers. During the diffusion process, the devices were placed into the incubator for 30 minutes, by the end of which the substrate concentration in the chamber will reach more than 90% (Supplementary Fig. 11). To isolate each culture chamber for proteolytic activity measurement, a positive pressure around 45 Psi, supplied by as air pump, was alternatingly applied to the inlet and outlet separately to remove the media in the main channel. The cells are incubated for one hour to allow the substrate to react with the proteolytic enzyme secreted by the cells. After a final wash step, fluorescence images were taken to readout the assay results. Since dead cells can release intracellular protease into the well, skewing the results, any cell with questionable viability was excluded from the analysis (Supplementary Fig. 23). After the assay, the neighboring chambers (upstream and downstream) of bright chambers (those that contain cells with high proteolytic activity) remain a similar fluorescent intensity as the rest of the empty chambers as shown in Supplementary Fig. 24, supporting negligible cross-contamination between chambers.

To perform the dynamic proteolytic activity assays, the aforementioned protocol was repeated every 8 hours for two days. After each measurement, media was pipetted into the device inlet and refilled into the main channel by applying negative pressure at the outlet. Any substrate remaining after each assay was able to diffuse out into the main channel during the 8 hours period so that the next measurement would not be interfered by the previous one (Supplementary Fig. 20).

### Image acquisition

The microfluidic chips were imaged using an inverted microscope (Nikon). The bright-field and fluorescent images were taken with a 10× objectives and a charge-coupled device (CCD) camera (Coolsnap HQ2, Photometrics). A FITC filter set was used for the fluorescent imaging. The microfluidic cell chamber array was scanned with a motorized stage (ProScan II, Prior Scientific). Before each scanning, the stage was leveled to ensure the image remained in the focus throughout the whole imaging area.

### Single cell sphere formation

To form single cell derived sphere, cells were loaded into a single cell suspension culture environment. Cells were first harvested from a petri-dish with 0.05% Trypsin/EDTA and centrifuged at 1000 rpm for 5 minutes. Then, cells were re-suspended at 0.5 × 10^6^ cells/mL in culture media, and 100 μL of the cell solution was injected into the inlet of the assay chip. The flow (0.04 μL/min) is generated spontaneously by the liquid height difference (5 mm, 50 Pa pressure) between the inlet and the outlet, so no external pump is required. Within 5 minutes, the cells were hydrodynamically captured in each chamber at single cell resolution, and the cell solution was replaced with serum-free culture media. For sphere formation assays, cells were cultured in serum-free MEBM (CC-3151, Lonza) supplemented with B27 (Gibco 17504-044), 20 ng/ml bFGF (BD 354060), 20 ng/ml EGF (BD 354052), 5 μg/mL insulin (Sigma I6634), 1 mM lipid concentrate (Gibco 11905-031), 1 μg/mL hydrocortisone (Sigma H4001), 100 μM mercaptoethanol (Sigma M3148), 10 μM cholesterol (Sigma C4951), and 1% penicillin/streptomycin (Gibco 15070). As the substrate is non-adherent, single cells grew in suspension to form spheres. The cells were monitored for 14 days, and spheres larger than 50 μm in diameter were harvested for use.

### Sphere retrieval, staining, and dissociation

After sphere formation, we retrieved the single cell derived spheres to investigate the intra- and inter- colony heterogeneity. First, 1 μM of blue fluorescent dye (Invitrogen, Cell tracker C2110) in PBS was flowed (0.04 μL/min) into the device for 3 minutes. Within this time period, only the outer cells in the sphere were thoroughly stained. Then, the device was dissembled and washed by 1 mL of PBS. A sphere of interest (90–100 μm diameter) was then retrieved with 1 uL of PBS into a tube. 3 uL of 0.05% Trypsin/EDTA (Gibco 25200) was added to the tube and pipetted up and down for 20 times to enhance the dissociation of the sphere. The tube was incubated for 5 minutes and then pipetted up and down for 20 times again. 3 μL of serum media was added into the tube to neutralize the trypsin. This cell mixture was then loaded into the chip for proteolytic analysis. After cell loading, the serum free media was used to wash the main channel 3 times, so the residual trypsin could be washed away. To distinguish the inner cells and outer cells, we set the threshold for distinguishing the two populations based on the average fluorescent intensity of all cells, so it is likely to have equal number of outer and inner cells for comparison.

### Data analysis and processing

The average fluorescent intensity of each cell chamber was quantified using Nikon Research Basics software. To analyze the proteolytic activity of single cells, the fluorescent intensity of each chamber was normalized to the chambers without cells in the same device. This procedure minimizes the device-to-device variation. The chambers that captured multiple cells, or that contained lysed cell and cell debris were not included for analysis. Two-tailed, unpaired student’s t-tests were used for all comparisons, with a significance level of 0.05 considered statistically significant. *Refers to P < 0.05, **Refers to P < 0.01, and ***Refers to P < 0.001. The data in the bar graphs (created using Excel) are presented as mean ± SD. The Box graphs were plotted using Origin 9.0. The bottom and top of the box are the first and third quartiles, and the band inside the box is always the second quartile (the median). The ends of the whiskers represent the 5^th^ percentile and the 95^th^ percentile. The square inside the box indicates the mean, and the x outside the box indicates the minimum and maximum of all of the data. For the dynamics of single cell proteolytic assay, we used R and R packages from CIMminer for hierarchical clustering. For clustering, we used Euclidean distance and Average Linkage. We used equal quantile as the binning algorithm to determine the colors assigned to values.

## Additional Information

**How to cite this article**: Chen, Y.-C. *et al*. Single Cell Proteolytic Assays to Investigate Cancer Clonal Heterogeneity and Cell Dynamics Using an Efficient Cell Loading Scheme. *Sci. Rep*. **6**, 27154; doi: 10.1038/srep27154 (2016).

## Supplementary Material

Supplementary Information

Supplementary Video S1

Supplementary Video S2

Supplementary Video S3
