# Supplementary material for: Single Cell Proteolytic Assays to Investigate Cancer Clonal Heterogeneity and Cell Dynamics Using an Efficient Cell Loading Scheme
Source: Sci Rep. 2016 Jun 10;6:27154. doi: 10.1038/srep27154 (PMC4901291; doi:10.1038/srep27154)
Supplement: Supplementary Information [file srep27154-s1.pdf]

Title: Single Cell Proteolytic Assays to Investigate Cancer Clonal Heterogeneity and Cell Dynamics Using An Efficient Cell Loading Scheme

Authors and affiliations:

Yu-Chih Chen[a, c^], Yu-Heng Cheng[a^], Patrick Ingram[b], and Euisik Yoon*[a, b]

[a]Department of Electrical Engineering and Computer Science, University of Michigan, 1301 Beal Avenue, Ann Arbor, MI 48109-2122;

[b]Department of Biomedical Engineering, University of Michigan, 2200 Bonisteel, Blvd. Ann Arbor, MI 48109-2099, USA

[c]University of Michigan Comprehensive Cancer Center, 1500 E. Medical Center Drive, Ann Arbor, MI 48109, USA

^Authors made equal contributions

*Corresponding author

Yu-Chih Chen

1301 Beal Avenue, Ann Arbor, MI 48109-2122, USA

Tel: 734-272-7113; E-mail: yuchchen@umich.edu.

Euisik Yoon

1301 Beal Avenue, Ann Arbor, MI 48109-2122, USA

Tel: 734-615-4469; E-mail: esyoon@umich.edu.

Supporting Figures

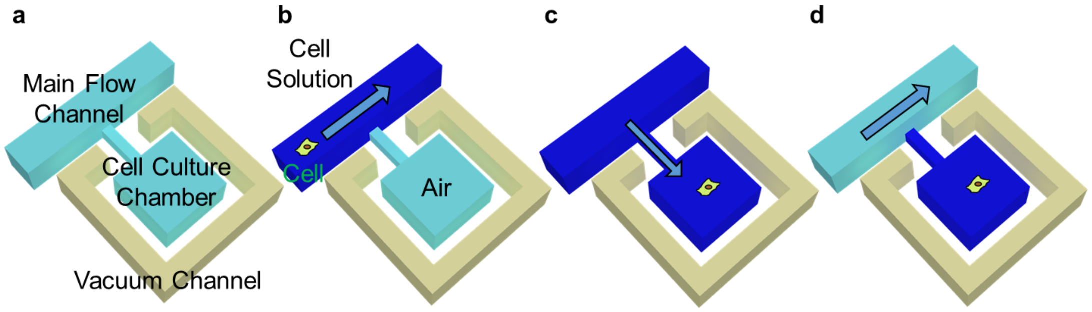

Figure S1. Vacuum driven single cell capture scheme. (a) The cell chamber is connected to the main channel and is surrounded by a vacuum channel. (b) In the beginning, the cell solution is driven into the main channel. (c) Vacuum is applied to the vacuum channel pulling the air through the PDMS and filling the chamber with the cell solution. (d) The cell solution is then driven further downstream to other chambers.

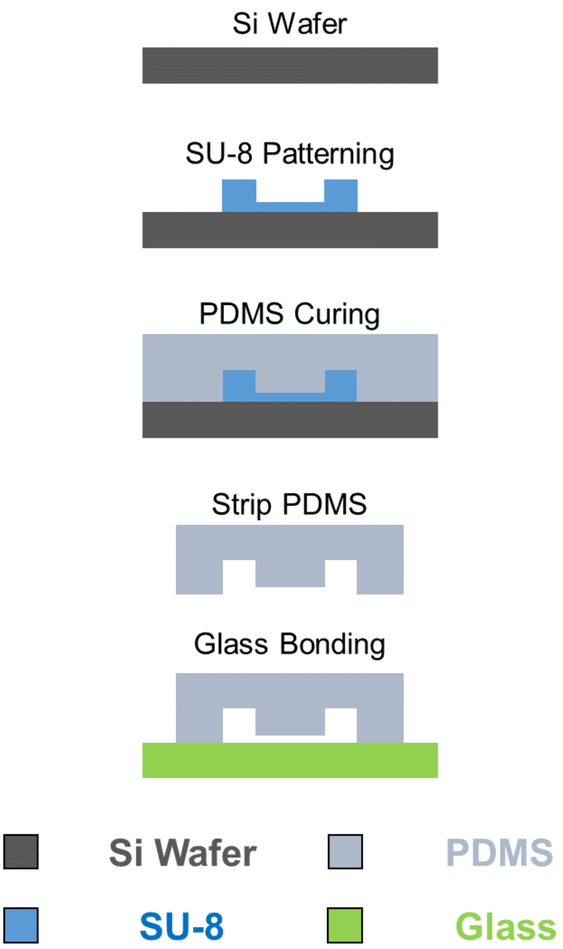

Fig. S2. Device fabrication processes. The device is composed of a PDMS layer bonded on a glass slide. First, and SU-8 master was created using photolithography on a silicon wafer. Then, the PDMS layer was fabricated using standard soft lithography processes, casting off the SU-8 master. Finally, the patterned PDMS was bonded to the glass after surface activation using oxygen plasma.

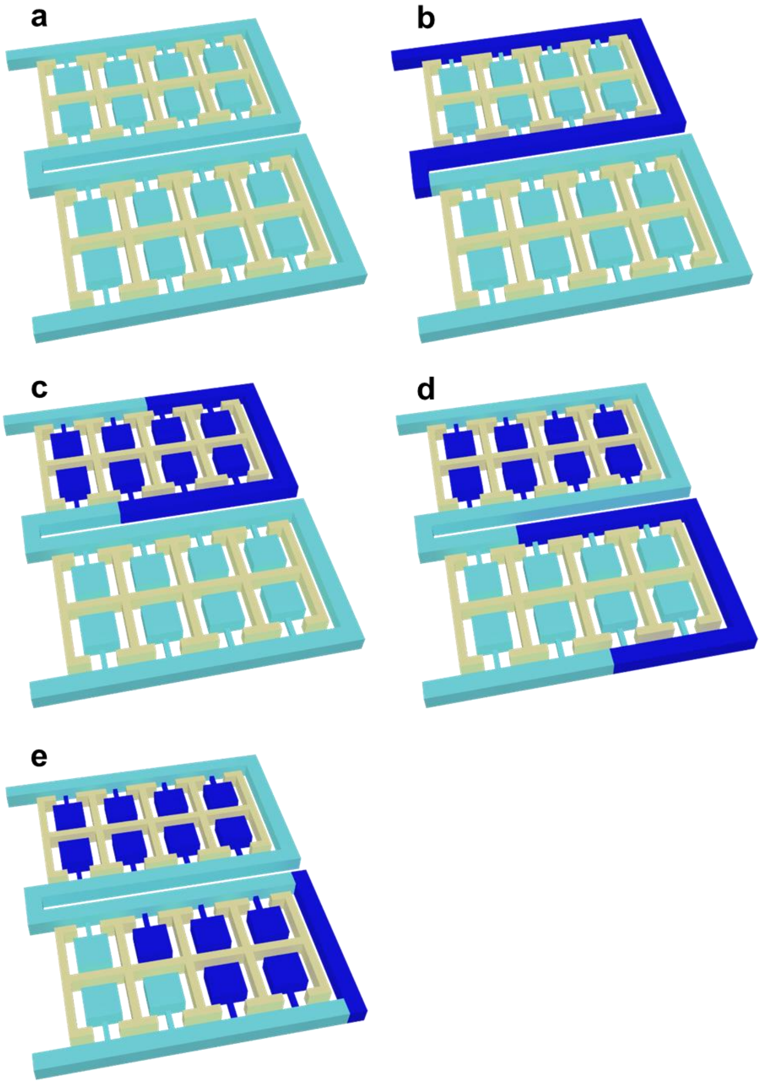

Figure S3. Serial high efficiency cell loading scheme. (a) The device consists of multiple rows of capture chambers that are loaded in a serial manner. (b) In the beginning, the cell solution is driven downstream into the first row. (c) Vacuum is applied to the vacuum channel, so the cell solution is driven into the chambers of the first row. (d) The cell solution is the driven further downstream to the second row of chambers. (e) Vacuum is applied to the vacuum channel, so the cell solution is driven into the chambers of the second row. After filling the chambers, the cell solution will be driven to the third row. In this manner, process is repeated until the total volume of the cell solution is loaded into the chambers of the device.

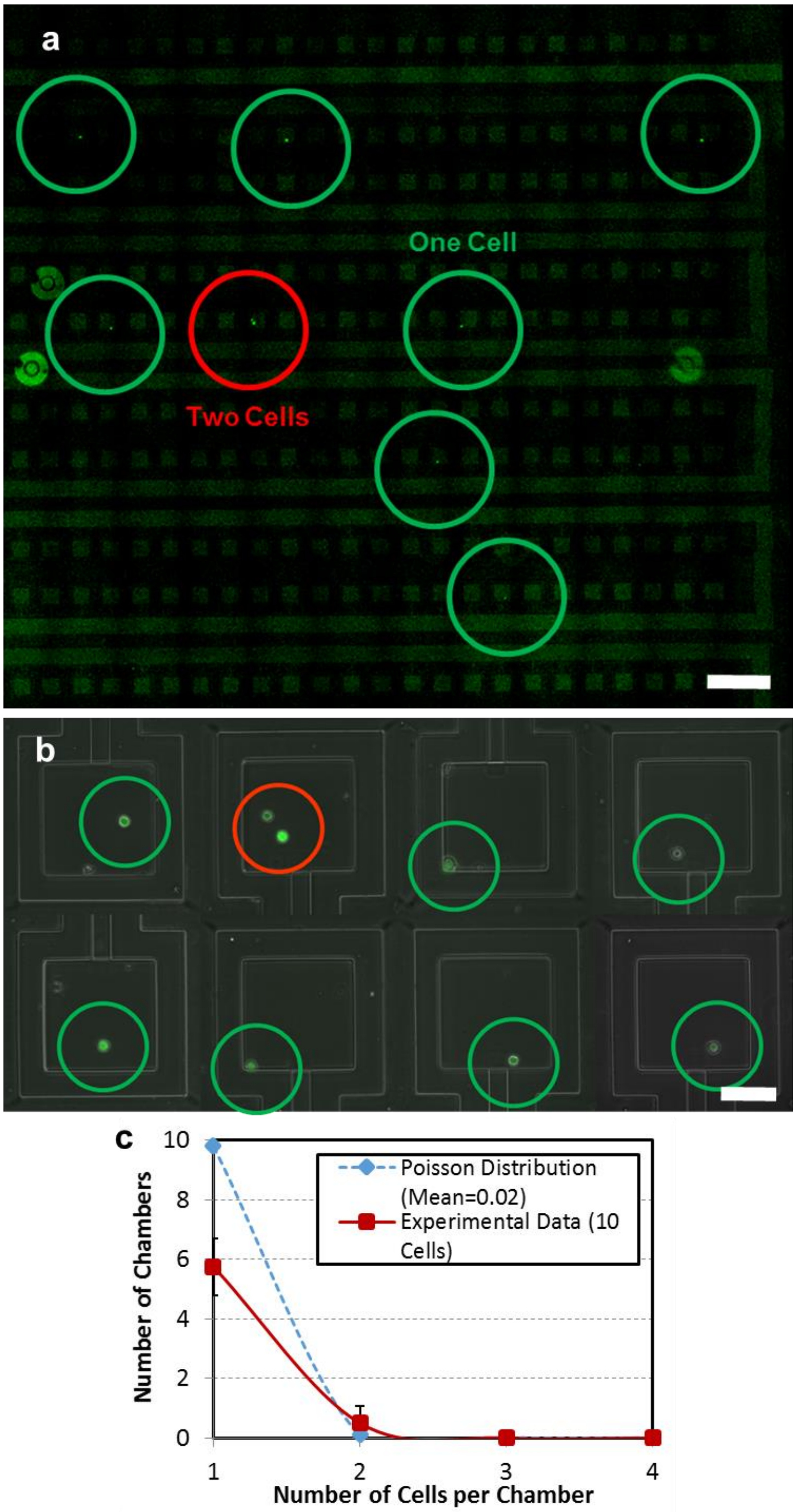

Figure S4. Cell capture of small samples (10 cells). (a) Fluorescent image of whole device (scale bar: 1 mm), (b) the enlarged view of chambers with captured cells (1-2 cells, scale bar: 100 µm), and (c) the Poisson distribution and the experiment results (N = 4 devices).

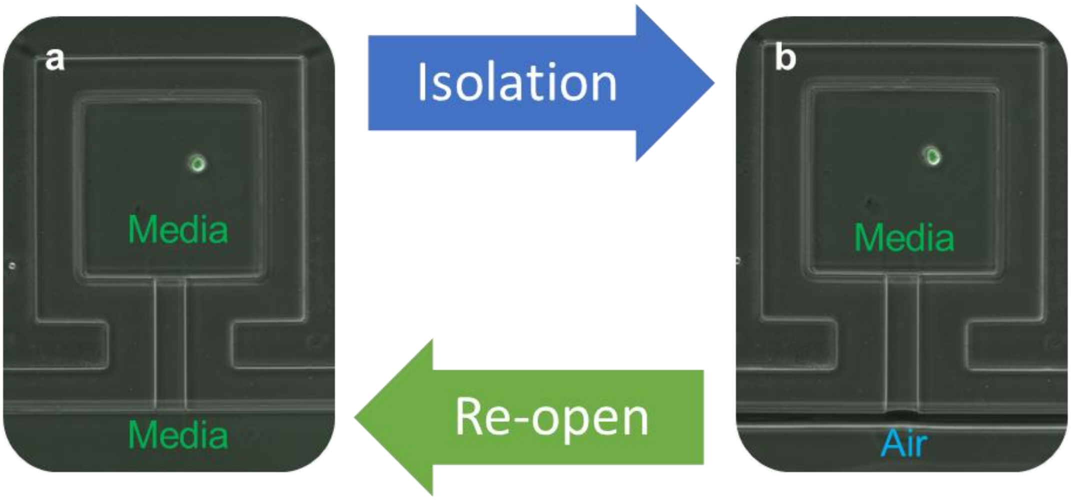

Figure S5. The isolation and re-opening of the cell chamber: (a) the chamber showing one cell captured before air isolation in the main channel and (b) after isolation.

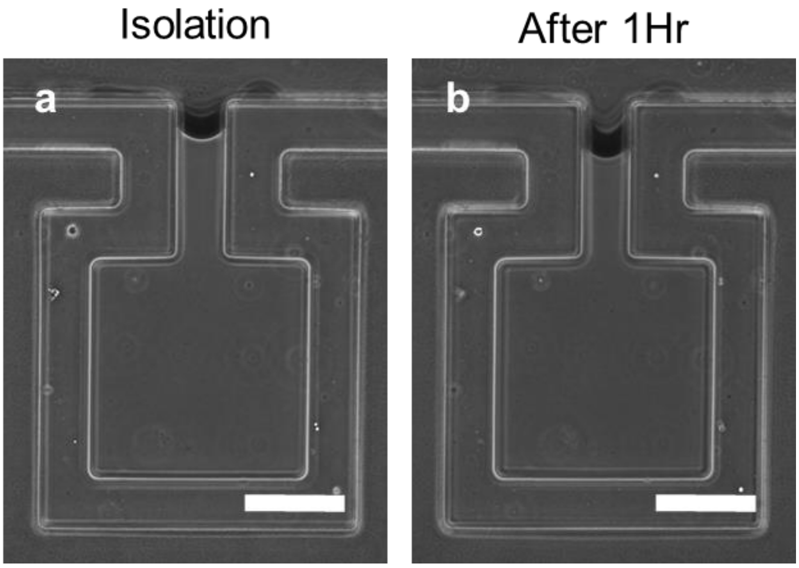

Figure S6. Evaporation of media during isolation. After one hour isolation, only 1-2% of the media has evaporated.

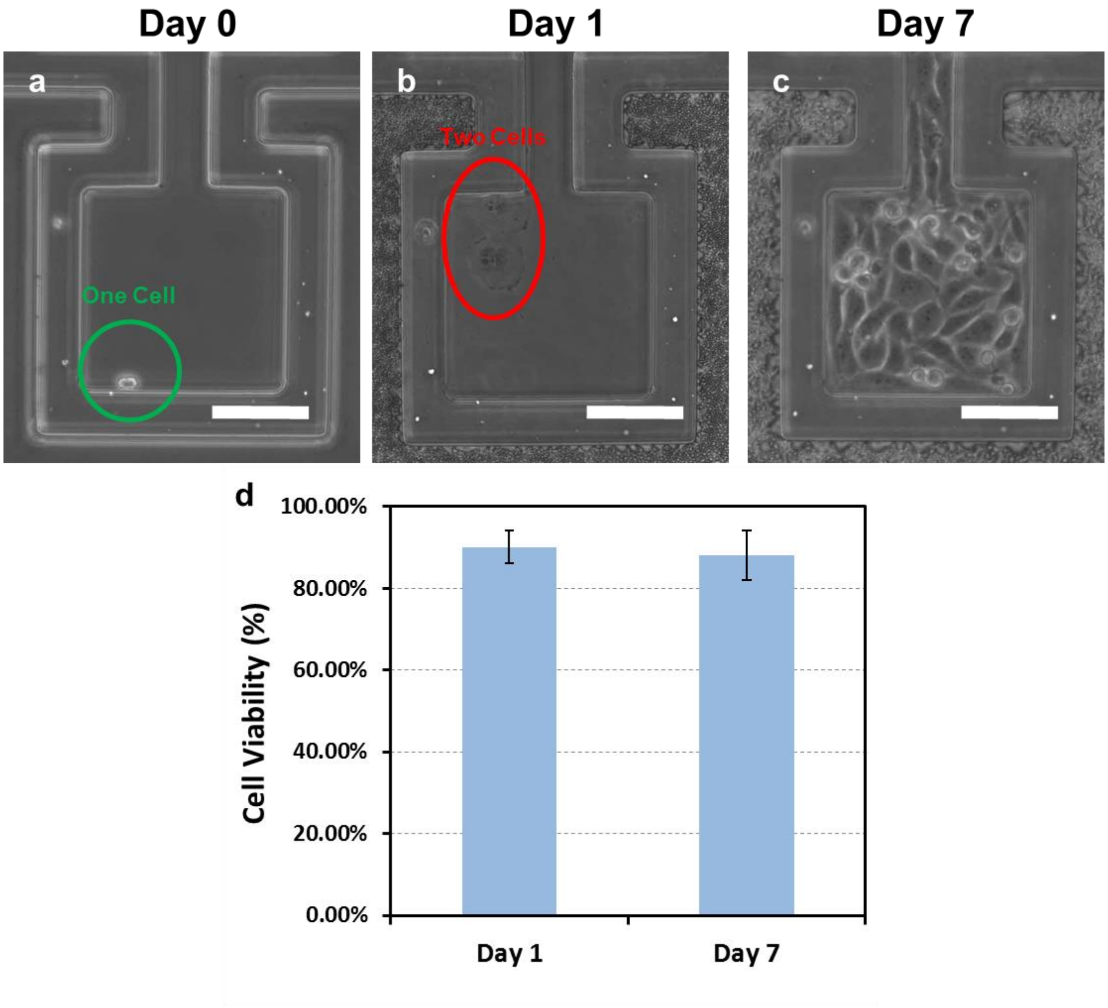

Figure S7. Cell viability of MDA-MB-231 in the chambers. (a) The cell chamber on Day 0 right after cell loading. (b) The cell chamber on Day 1. (c) The cell chamber on Day 7, showing high viability and proliferation on chip (scale bar: 100 μm). (d) The cell viability was maintained at 90% for over 7 days, demonstrating that the platform has little effect on cell viability. (N = 4 devices)

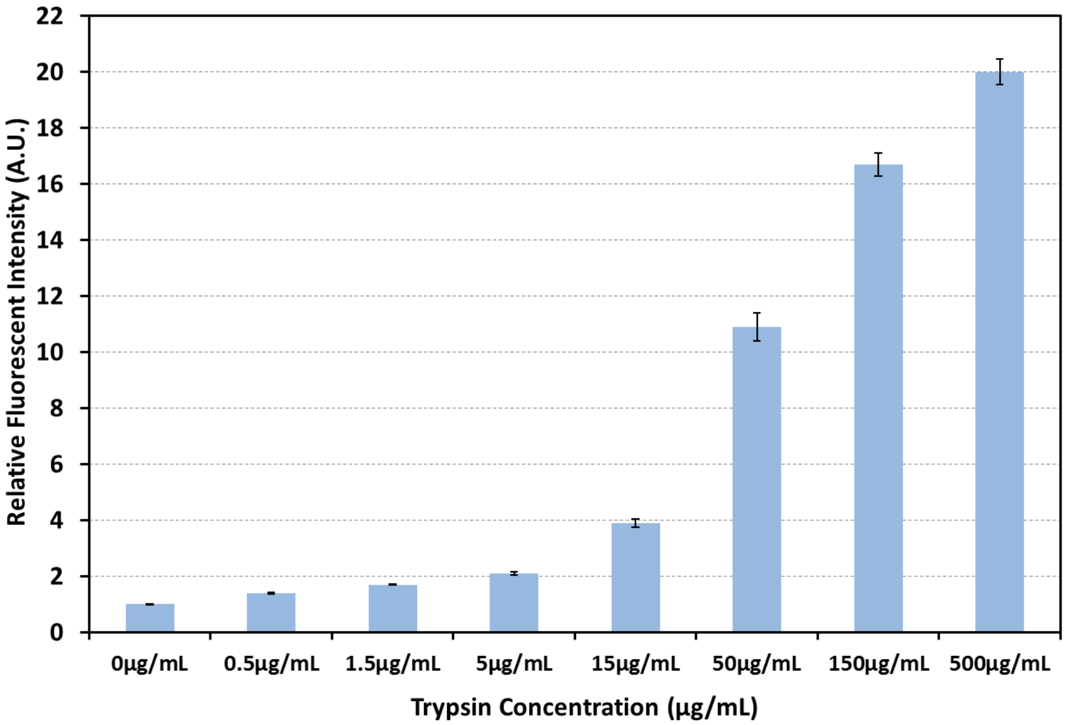

Figure S8. The fluorescent intensity of the substrate versus different concentration of trypsin (30 minutes reaction time), a protease that cleaves the FRET substrate. High concentration of trypsin cleaves more substrate, and the FRET signal becomes brighter. (N = 6 wells in 96-well plate)

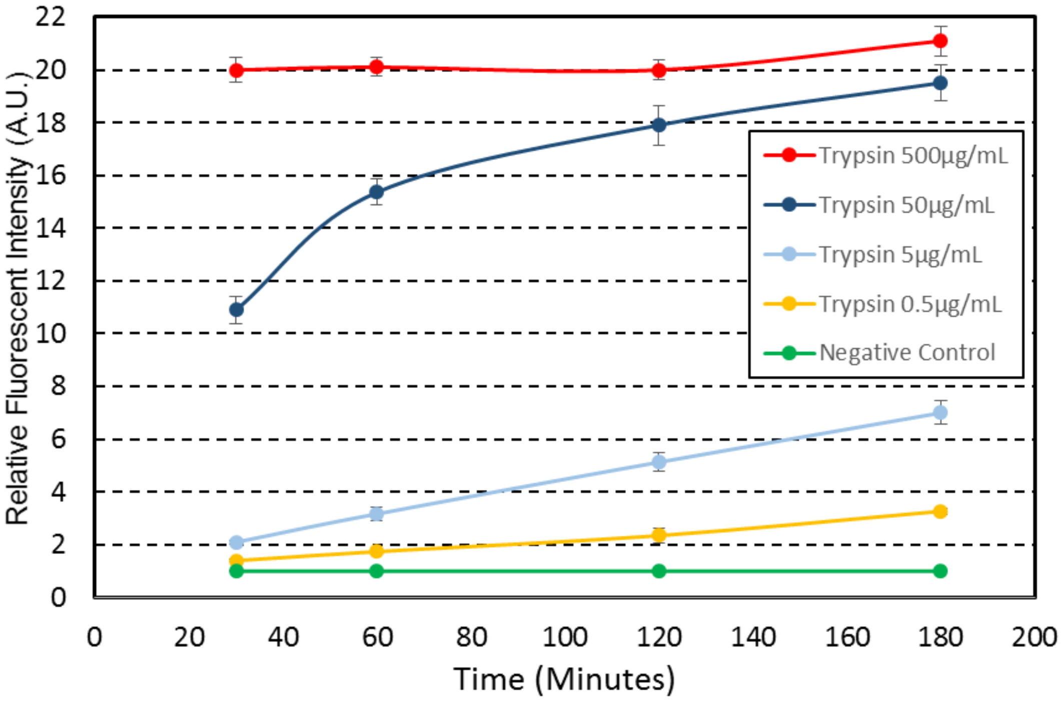

Figure S9. The fluorescent intensity of the substrate versus different reaction time. Under the conditions of low trypsin concentration, the fluorescent intensity increases

with increasing reaction time. Under the conditions of high trypsin concentration, the intensity saturates within the first 30 minutes. (N = 6 wells in 96-well plate)

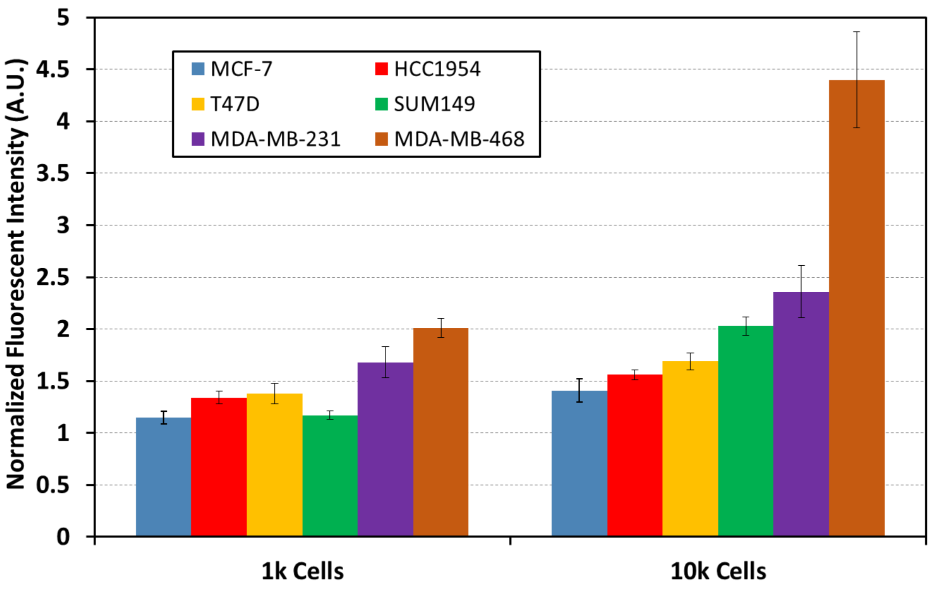

Figure S10. The fluorescent intensity of the substrate versus different number of cells and different cell lines (60 minutes reaction time). The fluorescent intensity was normalized to the well with no cell. The wells with more cells have higher proteolytic activity, and the MDA-MB-468 and MDA-MB-231 expresses higher proteolytic activity than other cell lines. (N = 6 wells in 96-well plate)

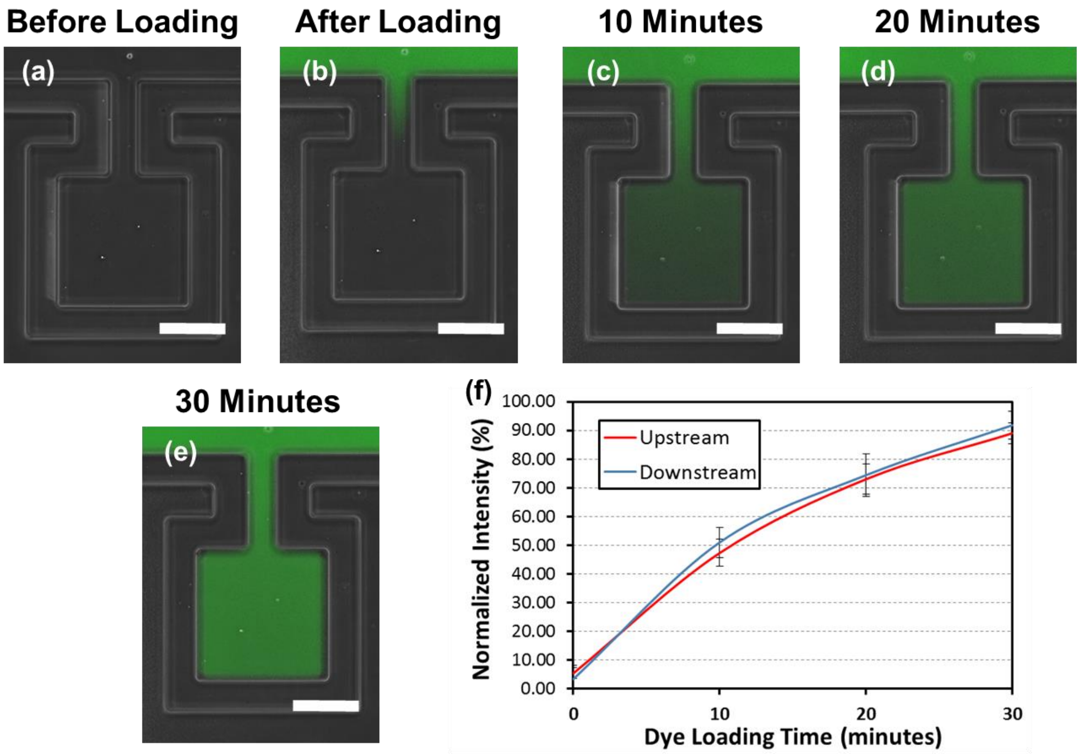

Figure S11. Diffusion of fluorescent substrate into the chamber. (a) Before loading of

the substrate. (b-e) 0-30 minutes after loading the substrate into the main channel. (f) The fluorescent intensity inside the chamber versus the time. (N = 10 chambers) The increase of the fluorescent intensity indicates the diffusion of the substrate into the chamber. The intensity in the chamber can reach 90% of that in the main channel within 30 minutes. (scale bar: 100 μm)

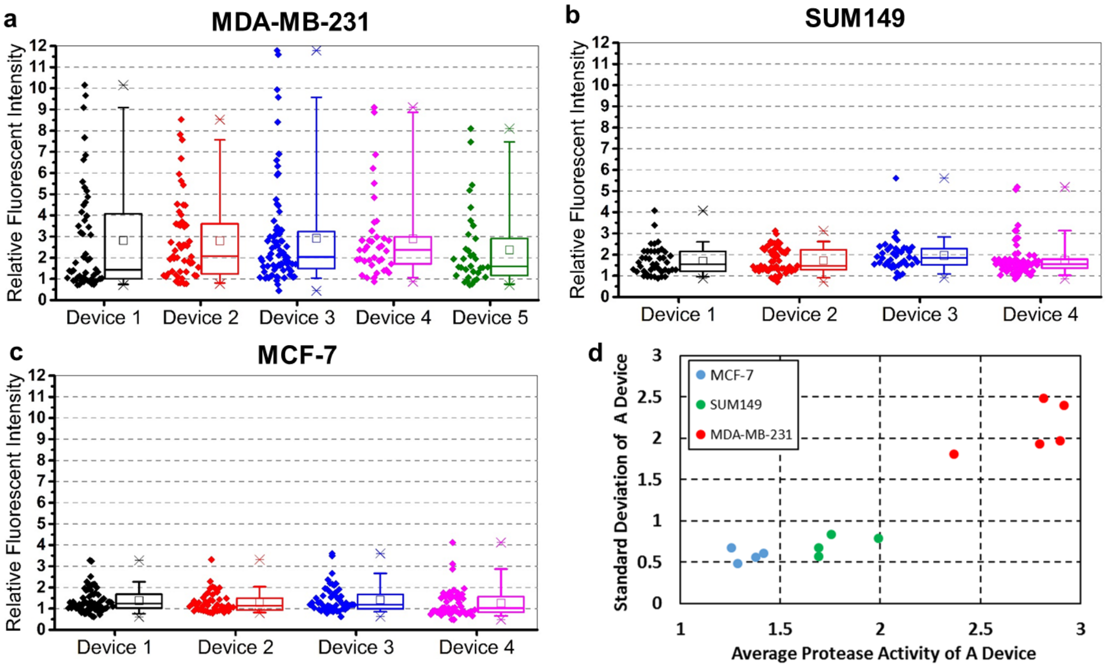

Figure S12. The assay variation between devices. The raw data of single cell proteolytic assays of (a) MDA-MB-231, (b) SUM149, and (c) MCF-7 cells. (N = 30-50 cells per device) (d) The distribution of devices: x-axis is the average of proteolytic activity, and the y axis is the standard deviation of cells in that device.

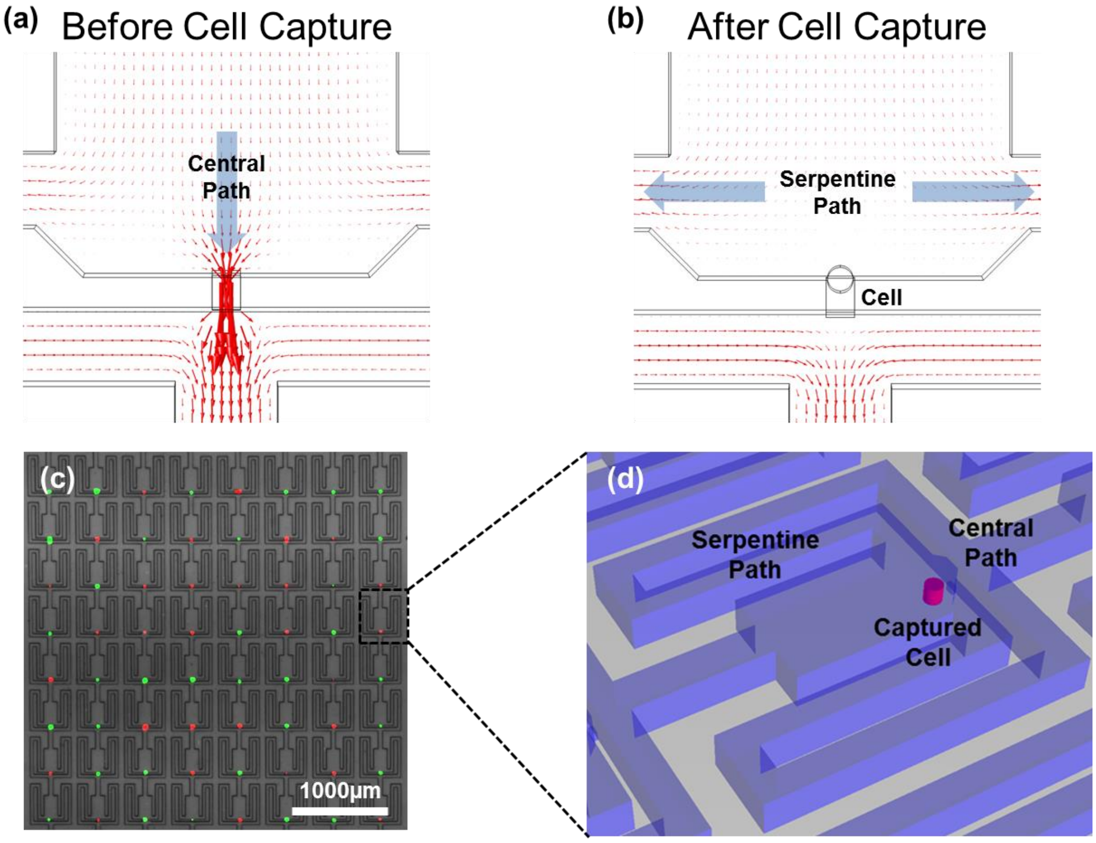

Figure S13. The hydrodynamic cell capture scheme. (a) The simulated (COMSOL 4.2) flow pattern before cell capture. The red arrows indicating flow direction and velocity suggest that the cells are likely to be guided to the capture site and get captured. (b) After cell capture, the captured cell blocks the flow, resulting in subsequent cells being guided into the serpentine path. (c) An 8x8 array of captured single cells. Two cell lines (red and green were different cell lines). were mixed for the demonstration. (d) Enlarged schematic of a cell capture chamber.

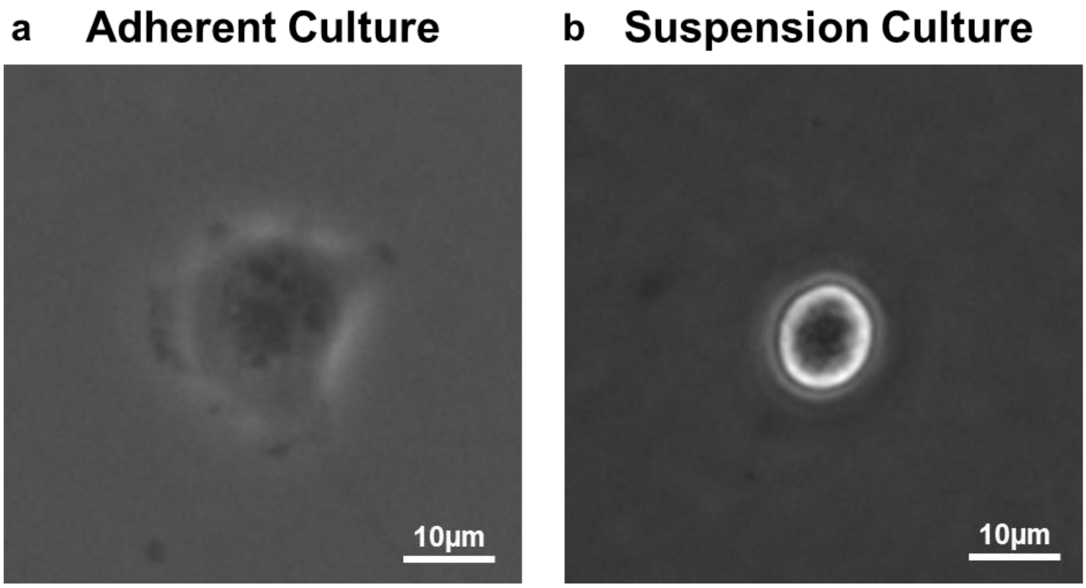

Figure S14. The comparison between (a) adherent culture of MDA-MB-231 on glass and (b) suspension culture of MDA-MB-231 on polyHEMA coated glass.

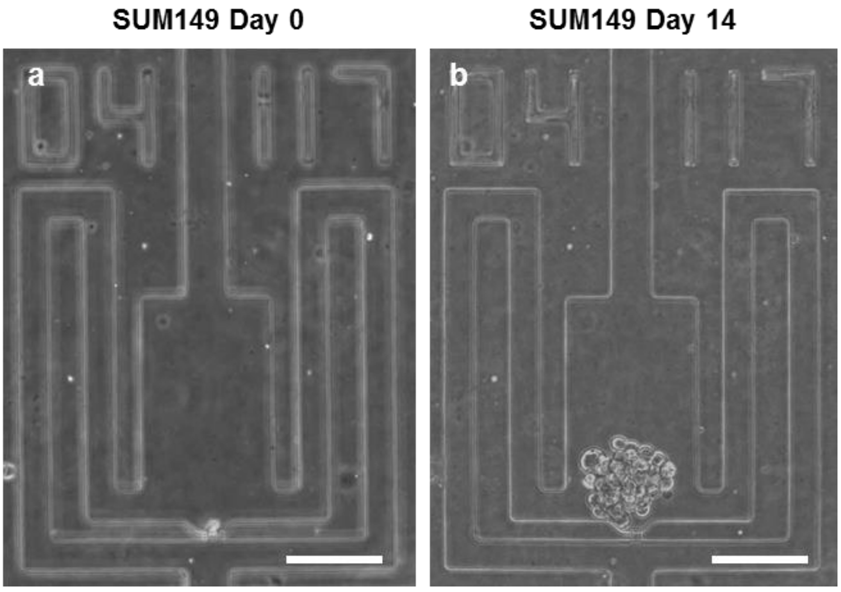

Figure. S15. The sphere formation of SUM149 in the single cell suspension culture chip. (a) Right after cell capture, we have single cell captured. (b) 14 days after cell loading, the single cell grew to a sphere.

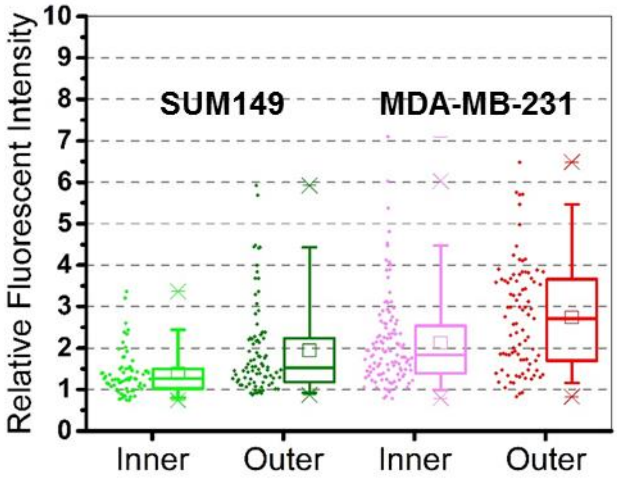

Figure S16. The comparison between inner and outer cells of all spheres (N ~ 100 for all conditions). Although the outer cells have higher proteolytic activity, the difference is less significant than comparing the differences observed between the spheres.

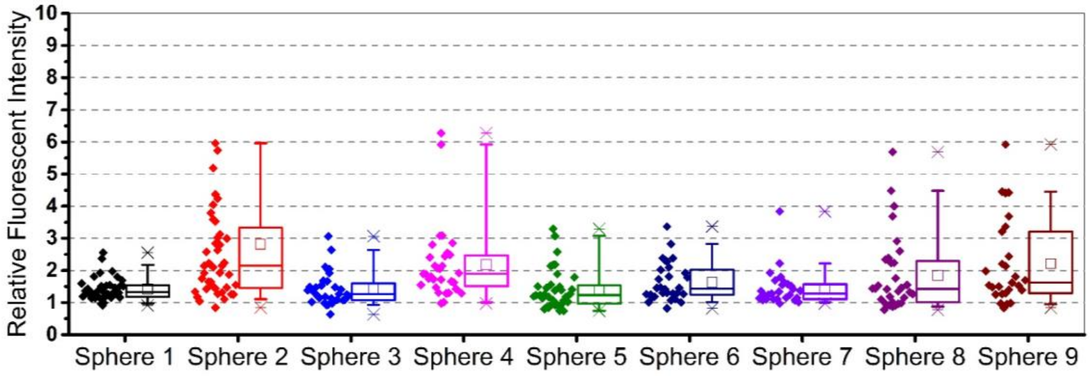

Figure S17. The raw data of single cell proteolytic activity from MDA-MB-231 spheres. (N = 30-50 cells per sphere)

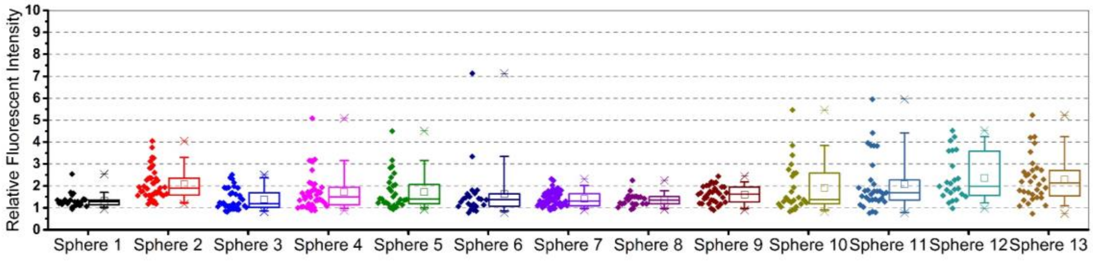

Figure S18. The raw data of single cell proteolytic activity from SUM149 spheres. (N = 30-50 cells per sphere)

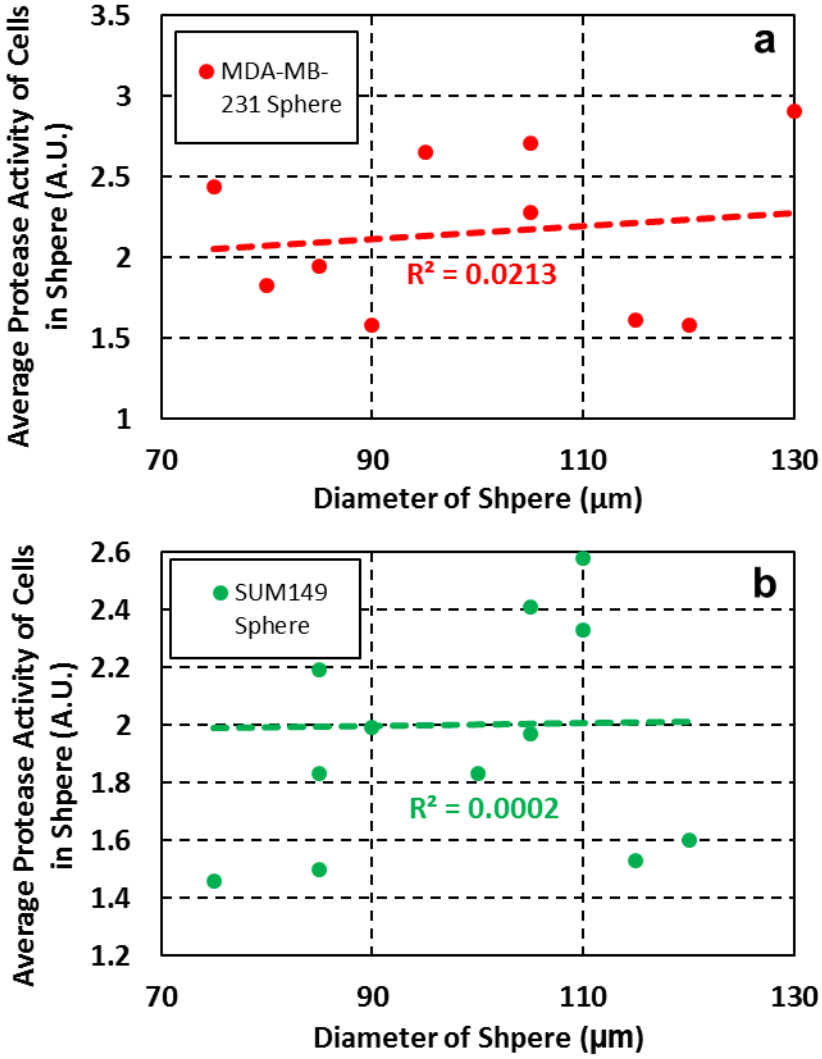

Figure S19. The correlation between the size of (a) MDA-MB-231 (N = 10 spheres) and (b) SUM149 (N = 14 spheres) spheres, and the proteolytic activity of single cells in that sphere. No correlation was observed.

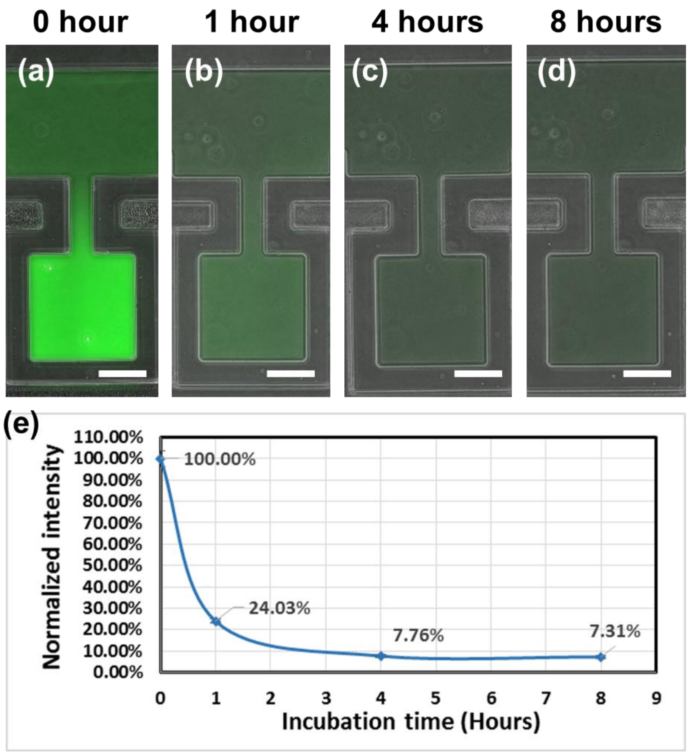

Figure S20. Diffusion of fluorescent substrate out of the chamber. (a) Chambers with substrate right after refilling culture media in the main channel. (b-d) 1, 4 and 8 hours after the media refill. (e) The fluorescent intensity inside the chamber versus the time. The decrease of the fluorescent intensity shows the diffusion of the substrate out of the chamber. The intensity in the chamber can be reduced to below 10% for the next proteolytic assay after 4 hours (N = 12 chambers). (scale bar: 100 μm)

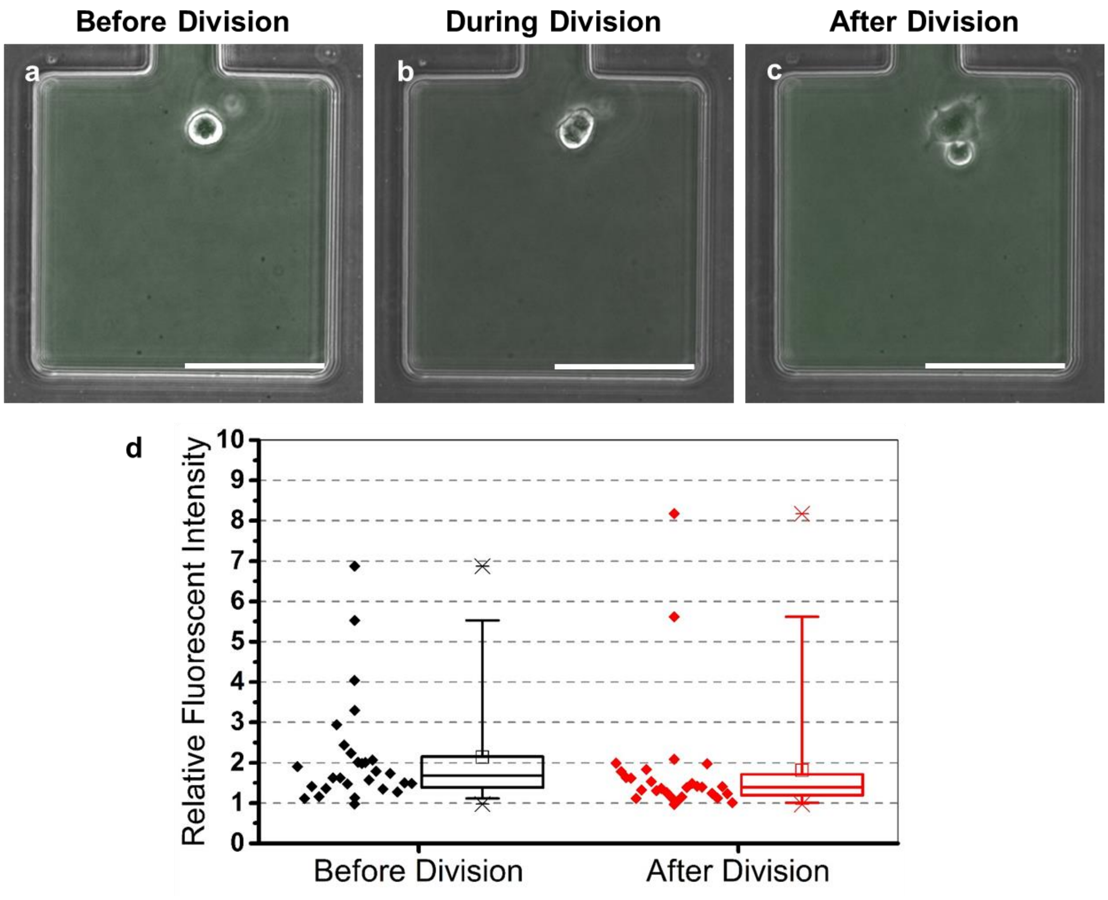

Figure S21. Cell division and proteolytic activity: (a) cell before division, (b) cell during division, (c) cell after division, and (d) the proteolytic activity of cell before and after division. (N = 20-30 cells per condition)

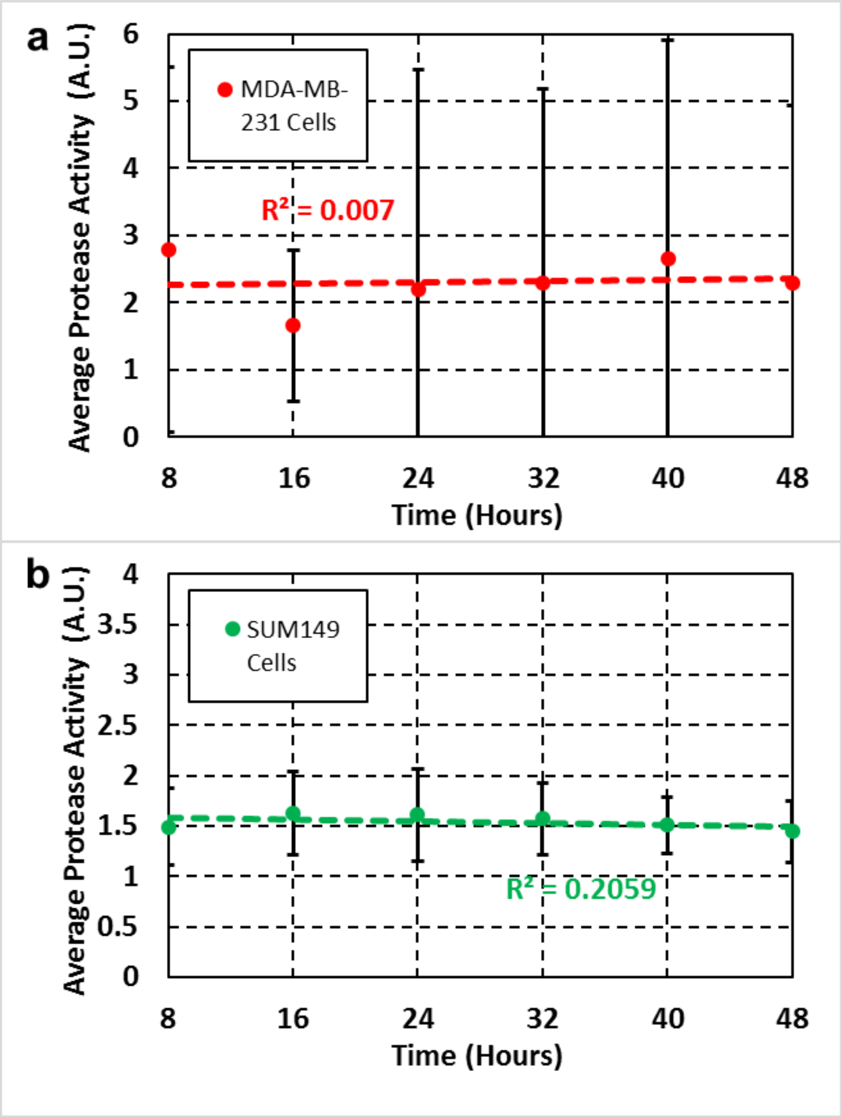

Figure S22. Average proteolytic activity of cells at different time points: (a) MDA-MB-231 and (b) SUM149 cells. Low correlation between time and activity indicates the cell behaviors didn't change or degrade over time. (N ~ 50 cells)

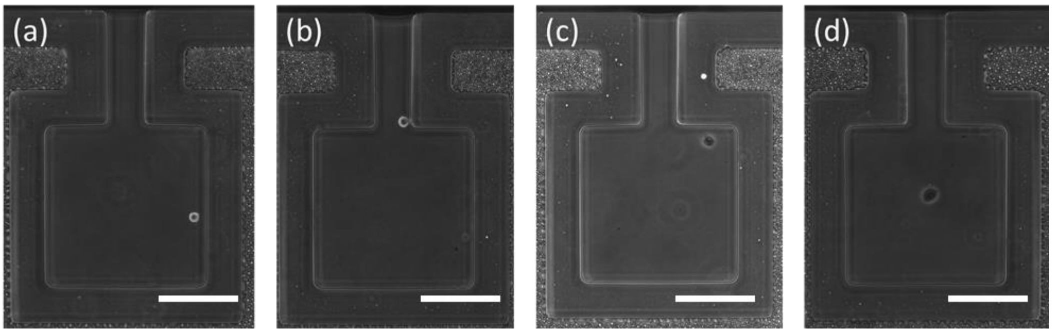

Figure S23. Cells captured in the microchambers: example (a, b) live cells and (c, d) dead cells. (scale bar: 100 µm)

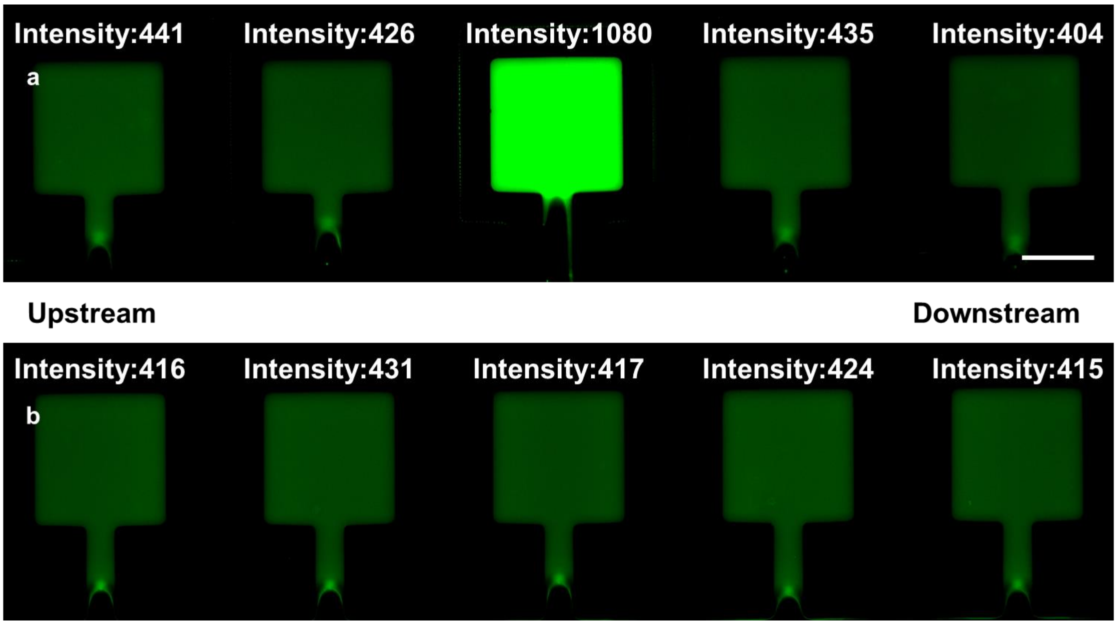

Figure S24. Chamber fluorescent intensity of (a) 4 empty (without cell) neighboring (2 in the upstream, and 2 in the downstream) chambers of a bright chamber (containing a cell of high proteolytic activity) and (b) 5 empty chambers in a row. The empty neighboring chambers have similar fluorescent intensity as the rest of empty chambers (scale bar: 100 µm)

Supplementary Video

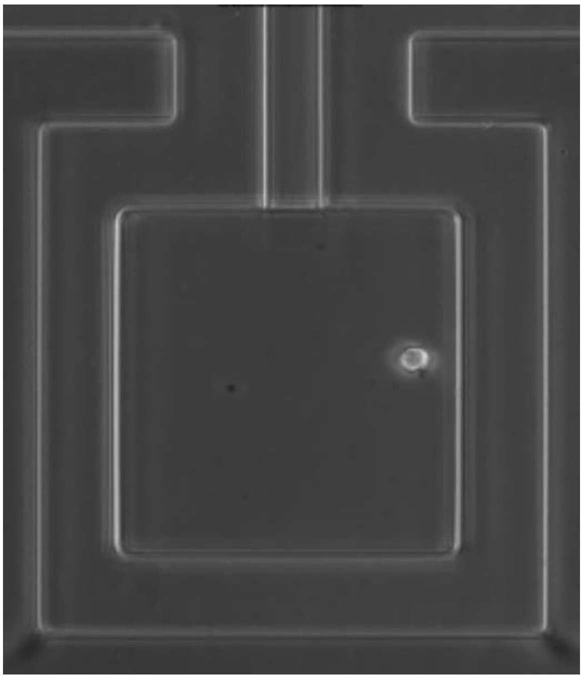

Supplementary Movie 1. The cell loading process of the high-efficiency loading scheme. A MDA-MB-231 cell was driven into the chamber by applying vacuum to the vacuum channel. After successful loading in the first location, the cell solution in the main channel was driven to the downstream for loading more chambers.

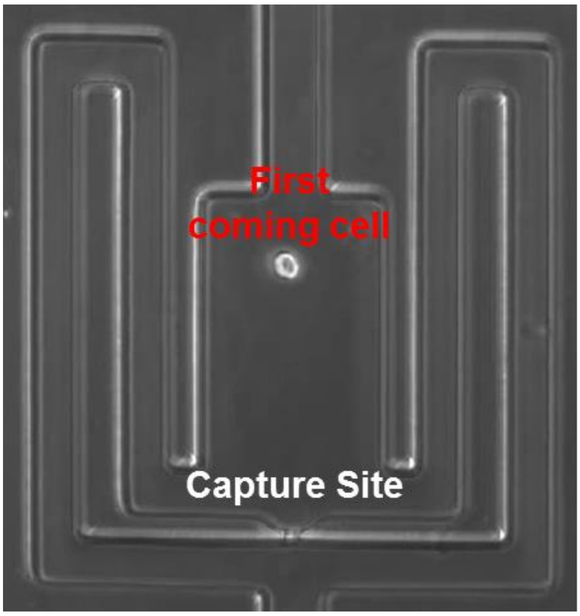

Supplementary Movie 2. MDA-MB-231 cells were loaded into the chamber by gravity flow, and the first cell was captured at the capture site.

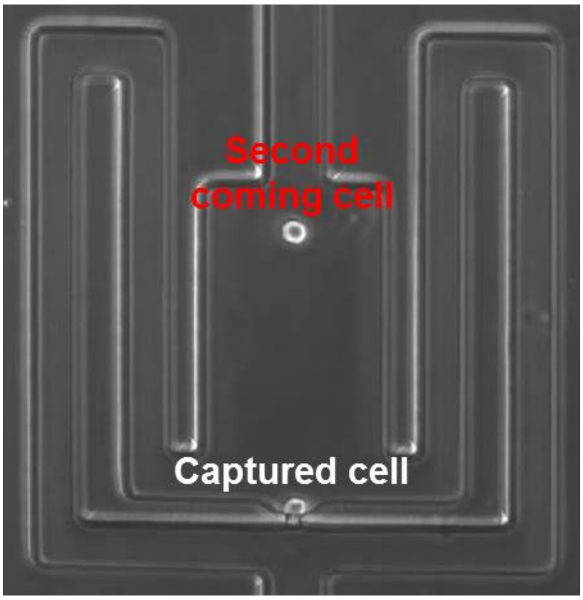

Supplementary Movie 3. MDA-MB-231 cells were loaded into the chamber by gravity flow. As the capture site was occupied by a captured cell, and the next coming cell flowed downstream through serpentine path.

Supplementary Note 1
SU8 Master Fabrication Protocol
Mask

1. Create the layout of the microfluidic chip using AutoCAD.
2. Load the 5”× 5” mask plate into the photomask fabrication tool, Heidelberg uPG 501.
3. Load layout file into the tool control computer and follow the file conversion procedure according to manufacturer’s manual to pattern the photomask by LED light exposure.
4. Develop the photoresist on the mask plate in MF-319 for 1 minute.
5. Etch the chrome on mask plate in Chromium Etch 1020 for 2 minutes.
6. Soak the mask plate in DI water for 5 minutes and dry it with compressed nitrogen.

Wafer clean

7. Place the wafer on hotplate at 80°C and blow supercritical CO_2 to the wafer.
8. Apply plasma treatment to the wafer surface with 100 sccm O_2 and 12 sccm Ar at 200 mT with 300W for 120 seconds.
9. Dehydrate the water for 5 minutes at 150°C

SU8 photolithography

10. Pour SU8 2010 resist on the wafer
11. Spread the resist at 500 rpm for 30 seconds. This step may be repeated multiple times until the resist fully cover the wafer surface.
12. Spin at 3000 rpm to achieve 100μm thick SU8 film

13. Perform the soft bake for 5 minutes at 65°C, 20 minutes at 95°C, and 5 minutes at 65°C.
14. Expose the wafer for 60s using MA/BA-6 Mask/Bond Aligner with optical filter to eliminate the UV radiation under 350 nm. The aligner UV supply was calibrated to 20 mW/cm^2.
15. Perform the post-exposure bake for 5 minutes at 65°C, 10 minutes at 95°C, and 5 minutes at 65°C.
16. Develop SU8 in MicroChem's SU-8 developer bath for 8 minutes and rinse with SU8 developer for 15 seconds after taking out from the bath.
17. Rinse with IPA and dry the wafer with compressed nitrogen.
18. Perform hard bake for 15 minutes at 150°C.

Release agent coating

19. Place the wafer in a desiccator.
20. Load 1 mL of the release agent, 3,3,3-fluoropropyltrichlorosilane, into a disposable boat inside the desiccator. Since the release agent is highly toxic, this step must be perform in the hood with great caution.
21. Reconnect the desiccator to the vacuum hose and open the vacuum valve for 10 minutes.
22. Leave the wafer in the desiccator overnight after closing the vacuum valve.

Supplementary Note 2

Device Fabrication Protocol

PDMS soft lithography

1. Pour 40.0 g SYLGARD® 184 Silicone Elastomer (PDMS) Base and 4.0 g Curing Agent in a disposable weighing boat.
2. Mix the silicone elastomer liquid thoroughly with stirrer. A homogeneous distribution of bubbles indicates good mixing.
3. Place the mixed silicone elastomer in a vacuum chamber and open the vacuum valve for 30 minutes or until the bubbles disappear.
4. Place the SU8 master wafer in a 4 inch circular foil pan.
5. Pour the PDMS into the foil pan.
6. Apply compressed nitrogen gently to the PDMS to remove any bubbles generated in the pouring process.
7. Place the foil pan on the hotplate at 100°C overnight to cure the elastomer.
8. Use the razor blade to cut the cured PDMS part along the edge of the wafer.
9. Pull up the PDMS part from the SU8 master slowly.

Device Assembly

10. Cut PDMS part into different blocks.
11. Punch holes to create the inlet, outlet, and the vacuum port.
12. Apply Scotch tape to clean the PDMS surface.
13. Apply plasma treatment to activate the surface of the PDMS blocks and the glass substrates with 100 sccm O_2 and 20 sccm Ar for 60 seconds at 250 mT with power of 80W.
14. Bond the PDMS block onto the glass substrate.
15. Place the bonded PDMS device on the hotplate at 80°C for at least 15 minutes.
